# Supplementary material for: Dielectrophoresis reveals stimulus-induced remodeling of insulin granule subpopulations
Source: Biophys J. 2026 Apr 25;125(11):2718–30. doi: 10.1016/j.bpj.2026.04.029 (PMC13352017; doi:10.1016/j.bpj.2026.04.029)
Supplement: Document S1. Figures S1–S11 [file mmc1.pdf]

Biophysical Journal, Volume *125*

Supplemental information

Dielectrophoresis reveals stimulus-induced remodeling of insulin granule subpopulations

Ashley Archambeau, Teji Korma, Aneesh Deshmukh, Mark A. Hayes, and Kate L. White

SUPPLEMENTAL INFORMATION

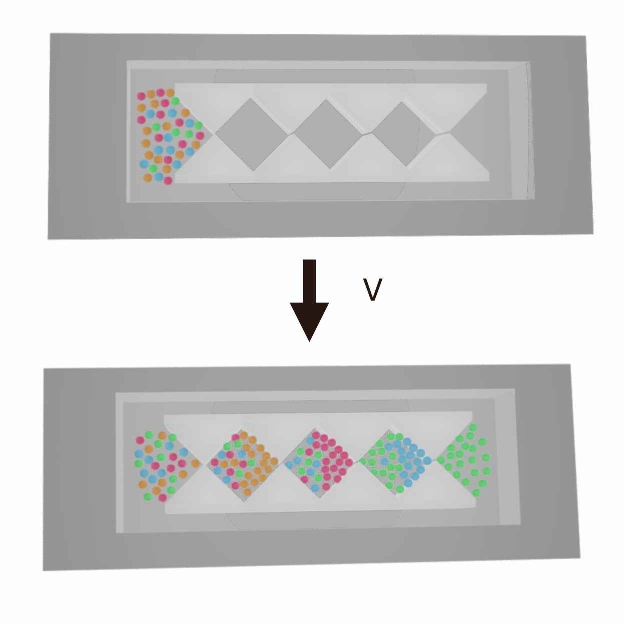

Figure S1. A truncated model of the microfluidic channel showing the separation of heterogeneous particles before (top) and after applying a voltage to separate particles (bottom).

A

Analyze intensity for each gate

EKMr (10^9 V/m^2)	Gate	Intensity
**1.40**	**1**	0.128
**1.40**	**2**	0.0842
**1.40**	**3**	0.0202
**4.10**	**4**	0.127
**4.10**	**5**	0.0467
**4.10**	**6**	0.0520
…	…	
**22.3**	**25**	0.0343
**22.3**	**26**	0.0299
**22.3**	**27**	0.0675

B

Average replicate values

EKMr (10^9 V/m^2)	Average Intensity	Scaled Intensity	
**1.40**	0.0775	0.284	
**4.10**	0.0753	0.275	
**6.70**	0.0602	0.217	
**9.30**	0.00428	0	**MIN**
**11.9**	0.0533	0.190	
**14.5**	0.163	0.616	
**17.1**	0.102	0.377	
**19.7**	0.262	1	**MAX**
**22.3**	0.0439	0.153	

C

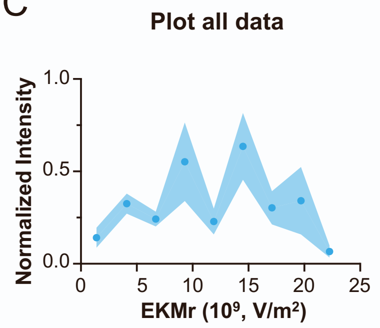

Figure S2. Example intensity processing and normalization workflow. A. The intensity of capture events is measured for each gate, with each technical replicate containing intensity measurements for each EKMr value in triplicate. Variation between these gates reflects sequential particle capture, as upstream gates are expected to capture more particles. Gates with no capture events were assigned a value of 0 before normalization. B. The intensity measurements for each EKMr value are averaged and assigned values of 0-1 according to the ratio of the intensity to the difference between the maximum and minimum intensity values. C. Each replicate is plotted to provide the typical distribution of a subpopulation. Shown here is the distribution of Rab3a at 1500 V, combining all biological replicates.

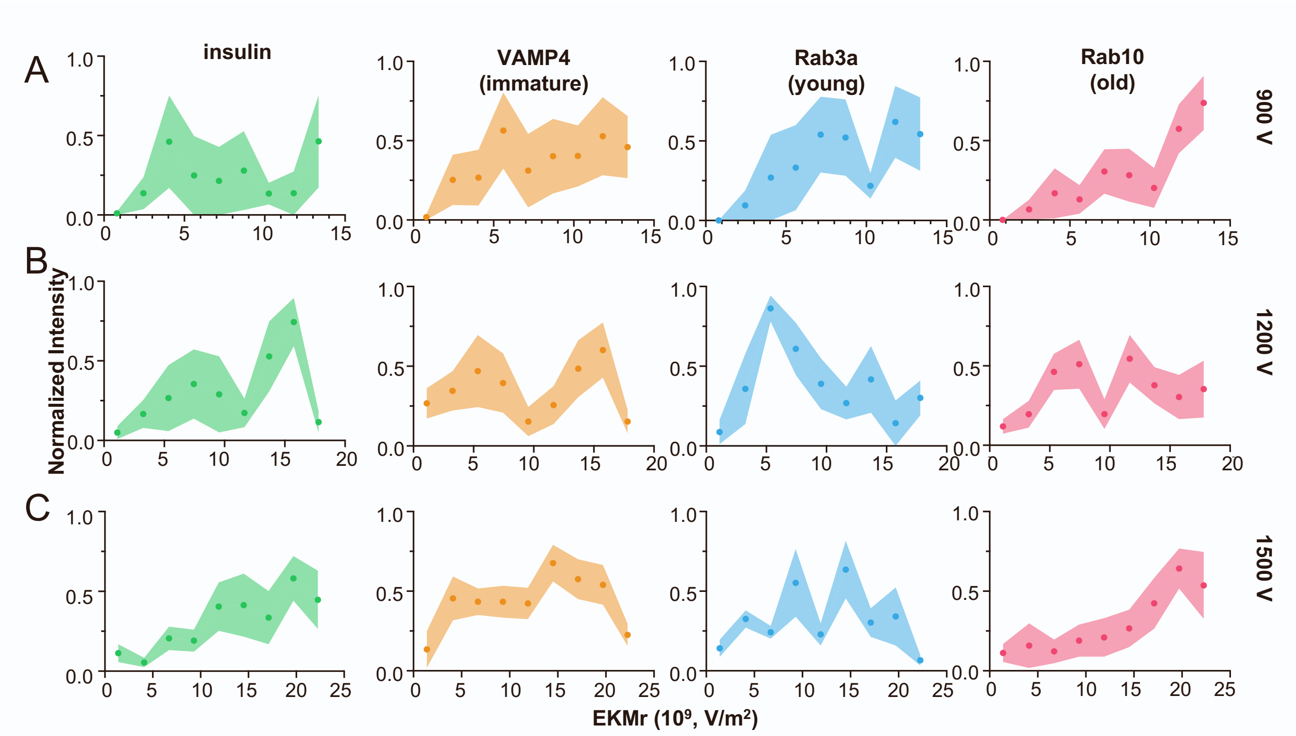

Figure S3. The EKMr distribution of different ISG subpopulations at **A.** 900 V (n=2-3 biologically independent experiments), **B.** 1200 V (n=3 biologically independent experiments), and **C.** 1500 V (n=3-4 biologically independent experiments). Values are mean ± SEM.

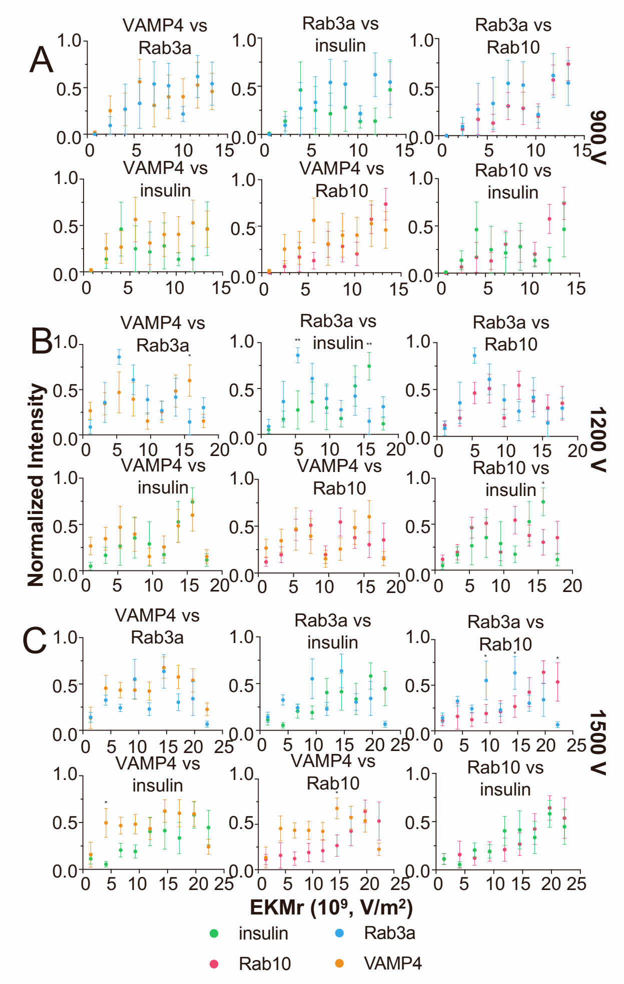

Figure S4. Comparisons of ISG subpopulation distributions at **A.** 900 V (n=2-3 biologically independent experiments), **B.** 1200 V (n=3 biologically independent experiments), and **C.** 1500 V (n=3-4 biologically independent experiments). Values are mean ± SEM (*p≤0.05, **p≤0.01 using ANOVA with Bonferroni post hoc multiple comparison correction).

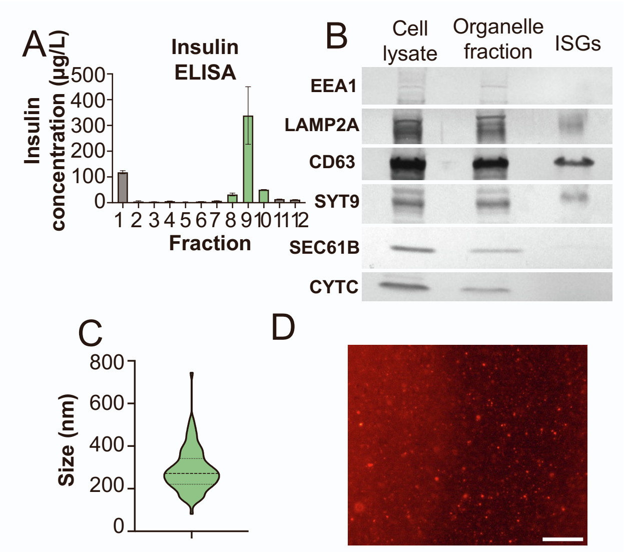

Figure S5. Validation of ISG isolation. **A.** Selection of fractions for use in separation experiments. Values are mean ± SEM. **B.** WB of cell lysate, organelle fraction, and isolated ISGs for determination of ISG purity. **C.** Size distribution of ISGs isolated from INS-1E cells. **D.** Fluorescence image of isolated ISGs. Scale bar: 250 μm.

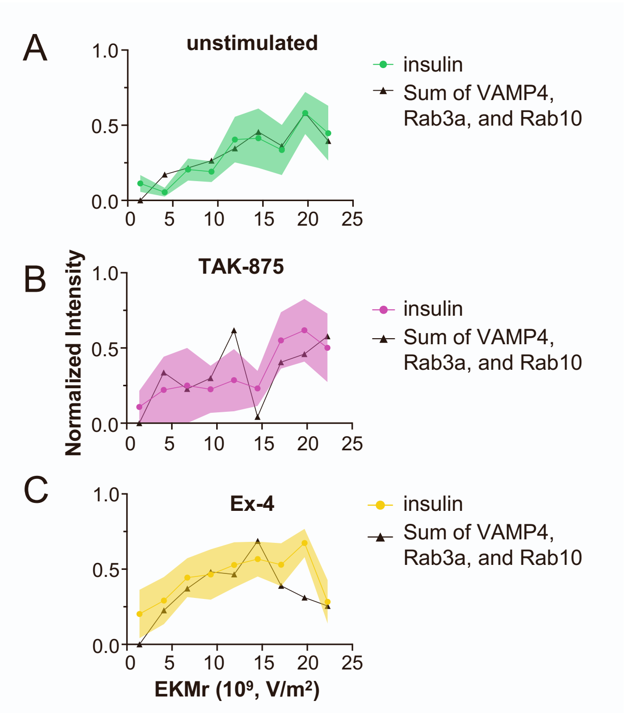

Figure S6. The cumulative subpopulation distribution approximates the total insulin distribution. For each EKMr bin, the independently normalized (0–1) intensities of VAMP4, Rab3a, and Rab10 were summed without weighting to generate a cumulative subpopulation signal. To facilitate visual comparison, this summed distribution was linearly scaled so that its maximum value matched the maximum of the corresponding insulin distribution within the same condition. Panels show **A.** unstimulated, **B.** TAK-875-stimulated, and **C.** Ex-4-stimulated conditions. Values are mean ± SEM, and lines connect points for visual guidance.

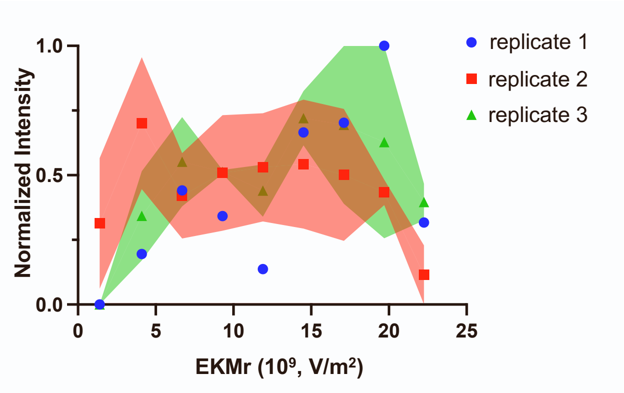

Figure S7. EKMr distributions of VAMP4-labeled ISGs separated at 1500 V from three independent biological experiments. Each replicate was independently background-corrected and scaled between 0 and 1 before plotting. Replicates exhibit similar overall EKMr profiles with expected biological variability across bins.

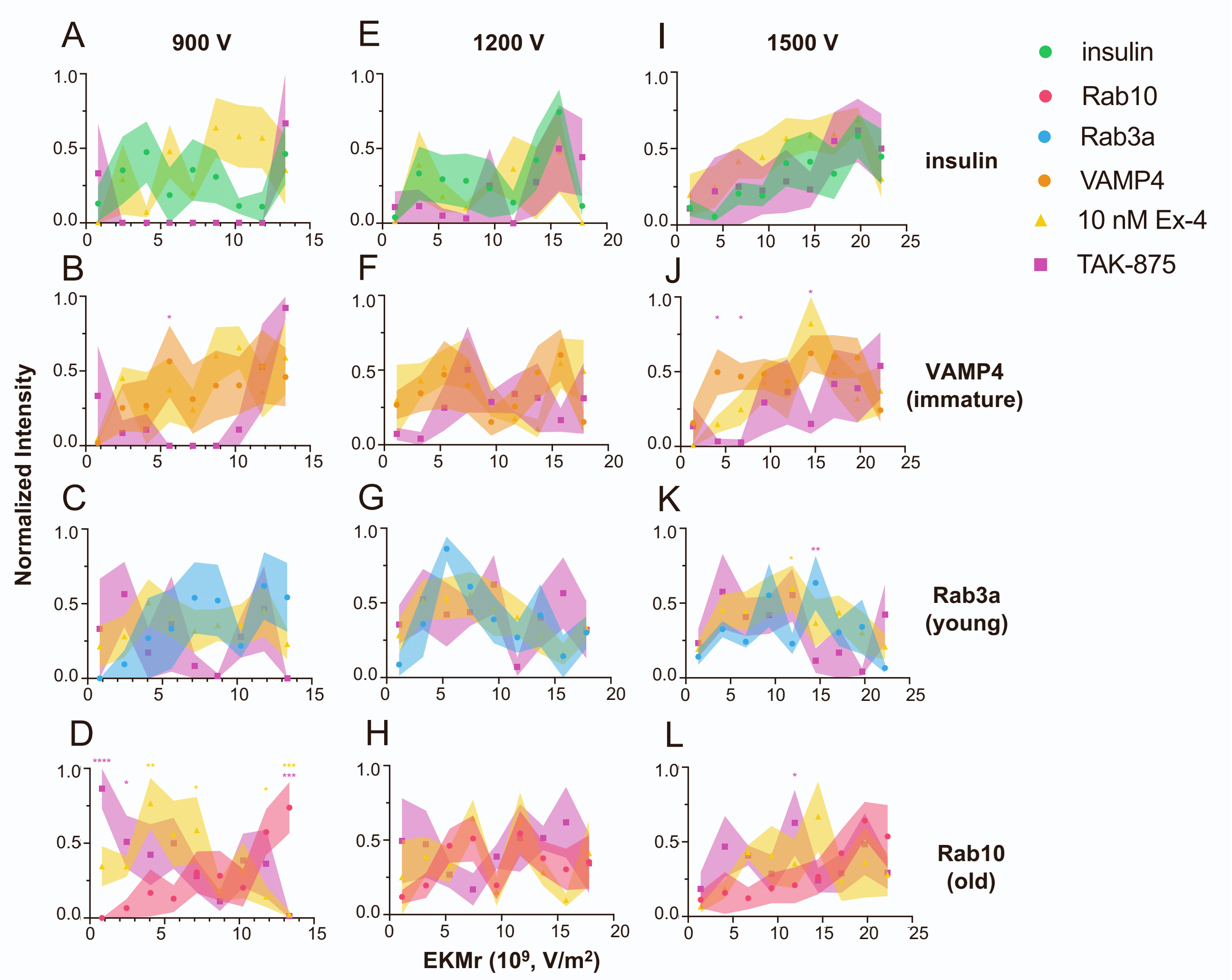

Figure S8. Changes in the EKMr distribution of each ISG subpopulation in response to TAK-875 and Ex-4 at **A-D.** 900 V (n=2-3 biologically independent experiments), **E-H.** 1200 V (n=2-3 biologically independent experiments), and **I-L.** 1500 V (n=2-4 biologically independent experiments). Values are mean ± SEM (*p≤0.05, **p≤0.01, ***p≤0.001 using ANOVA with Bonferroni post hoc multiple comparison correction. Comparisons made between stimulation and unstimulated conditions, symbols color-coded according to condition).

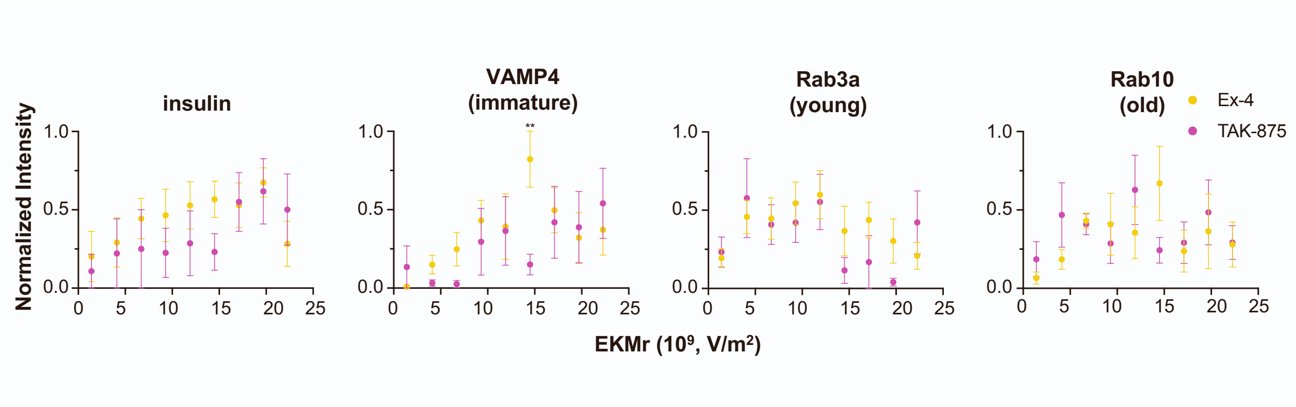

Figure S9. Differences in EKMr distributions between ISGs isolated from TAK-875 and Ex-4-stimulated cells at 1500 V (n=2-3 biologically relevant experiments). Values are mean ± SEM (*p≤0.05, **p≤0.01, ***p≤0.001 using ANOVA with Bonferroni post hoc multiple comparison correction).

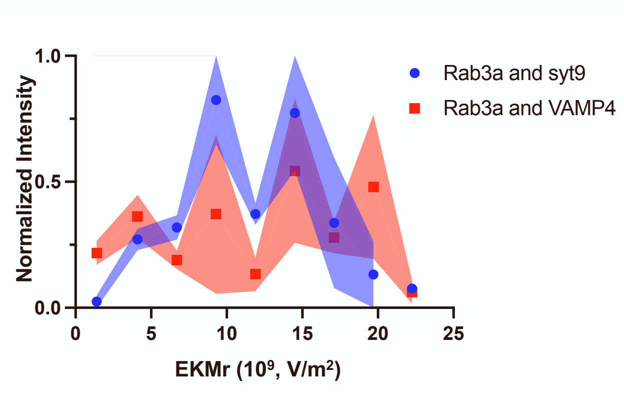

Figure S10. The EKMr distribution of young ISGs at 1500 V is not significantly affected by the differences in a second antibody label (syt9 or VAMP4, n=1-2 biologically independent experiments). Values are mean ± SEM.

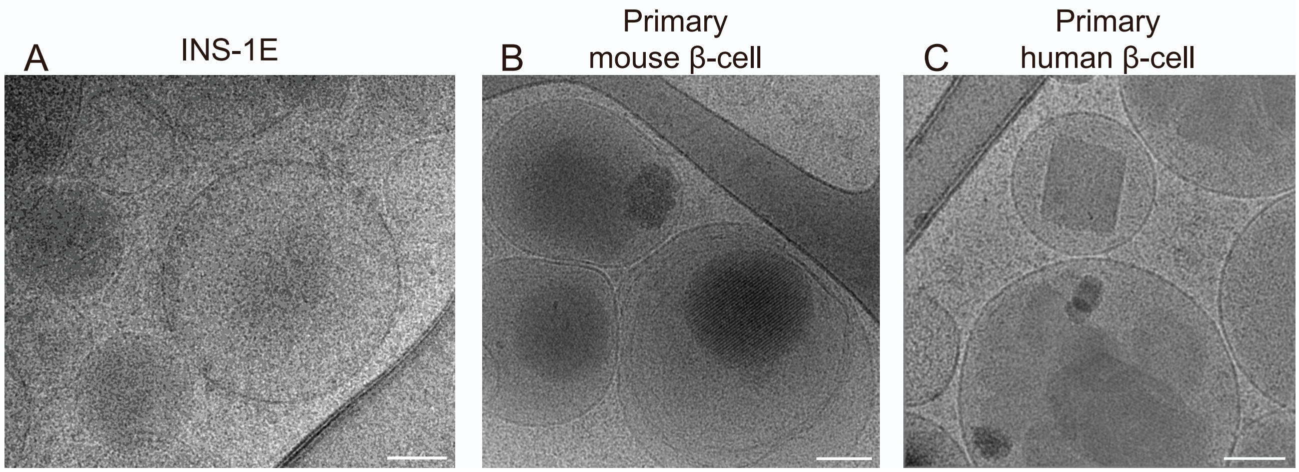

Figure S11. CryoET slices (**A-B**) and cryoEM images of ISGs inside INS-1E, primary mouse β-cells, and primary human β-cells. Magnification: **A-B.** 26,000x, **C.** 92,000x. Scale bar: 100 nm. A Gaussian blur of 1.0 nm was applied for clarity.

Table S1. Statistical comparisons between markers in the unstimulated condition.

Table S2. Statistical comparisons between the unstimulated condition and either the TAK-875 or Ex-4 condition.

Table S3. Statistical comparisons between the TAK-875 and Ex-4 conditions at 1500 V.

Video S1. Sample video of data collection of VAMP4-labeled ISGs separated in a microfluidic channel at 1500 V. Scale bar: 250 μm.
