# Supplementary material for: Dielectrophoresis reveals stimulus-induced remodeling of insulin granule subpopulations
Source: Biophys J. 2026 Apr 25;125(11):2718–30. doi: 10.1016/j.bpj.2026.04.029 (PMC13352017; doi:10.1016/j.bpj.2026.04.029)
Supplement: Document S2. Article plus supplemental information [file mmc4.pdf]

Biophysical Journal
Article

Biophysical Society

Dielectrophoresis reveals stimulus-induced remodeling of insulin granule subpopulations

Ashley Archambeau,[1] Teji Korma,[2] Aneesh Deshmukh,[1] Mark A. Hayes,[2] and Kate L. White[1,3,*]
[1]Department of Chemistry, Bridge Institute, USC Michelson Center for Convergent Bioscience, University of Southern California, Los Angeles, CA, USA; [2]School of Molecular Sciences, Arizona State University, Tempe, AZ, USA; and [3]Department of Quantitative and Computational Biology, USC Dornsife College of Letters, Arts, and Sciences, University of Southern California, Los Angeles, CA, USA

ABSTRACT The pancreatic β cell contains several functional subpopulations of insulin secretory granules (ISGs). These subpopulations vary in maturity, age, and secretory capacity. Differences in protein and lipid composition of ISGs are correlated with disease but require further study to understand how ISG remodeling regulates normal biology. Due to limitations in traditional separation methods, the extent of these subpopulations, any overlap between them, and how they are affected by insulinotropic signals have not been determined. In this work, we adapted direct current insulator-based dielectrophoresis (DC-iDEP) to separate ISGs isolated from INS-1E cells, an immortalized rat insulinoma cell line model, according to their electrokinetic mobility ratio (EKMr). We were able to separate ISG subpopulations from unstimulated cells to determine a baseline distribution and identify characteristic profiles for immature, young, and old ISGs. We then analyzed the distributions of subpopulations in cells stimulated with insulin secretion signals known to induce biophysical remodeling and maturation. We found significant changes in each subpopulation studied in response to stimulation, consistent with the increases in maturation and crystallization and changes in size reported in the literature. This work provides new insights into how the cell controls ISG remodeling and may drive future development of more effective therapies.

SIGNIFICANCE Understanding insulin secretory granule (ISG) heterogeneity and the functional role of subpopulations is a critical step toward unraveling the mechanisms of insulin secretion. We adapted direct current insulator-based dielectrophoresis (DC-iDEP) to resolve immature, young, and old ISG subpopulations based on their biophysical properties. We also explored the biophysical remodeling of ISGs under insulinotropic stimuli to further probe how the cell controls remodeling and secretion. Our work provides a new framework for quantifying granule heterogeneity by linking biophysical features to functional subtypes and assessing how environmental stimuli remodel ISGs. This methodology establishes a broadly applicable platform to interrogate organelle and vesicle diversity in complex biological systems.

INTRODUCTION

Insulin is a peptide hormone essential for blood glucose regulation.[1] It is produced by the pancreatic β cell and packaged into organelles called insulin secretory granules (ISGs), which then undergo a process called maturation in which they are prepared for secretion upon β cell stimulation.[2] ISG maturation involves the acidification of the ISG lumen, conversion of proinsulin to insulin, and insulin condensation or crystallization.[3,4] Due to the nature of this process, several ISG subpopulations with varying ages, cargo, secretory capacities, and levels of maturity are present in the β cell, but the extent of the heterogeneity of ISGs in the β cell is unclear.[5–7] Disease states of the β cell, such as diabetes, are associated with defects in maturation and changes in ISG subpopulation ratios.[6,8] As rates of diabetes rise worldwide, understanding insulin maturation and ISG heterogeneity is crucial.[9]

Several subpopulations have been identified. These subpopulations have been defined along multiple, overlapping axes, including functional differences, structural properties, and spatial arrangements within the cell. Functionally, ISG subpopulations have been described based on their secretion competence, calcium sensitivity, maturation state, and age.[5,6,10–14] Structurally, ISGs exhibit heterogeneity in size, protein and lipid composition, and pH, properties that are tightly linked to functional behavior and maturation state.[5,6,15–18] Spatially, ISGs vary in their proximity to the

Submitted October 24, 2025, and accepted for publication April 23, 2026.
*Correspondence: katewhit@usc.edu
Editor: Guy Genin.
https://doi.org/10.1016/j.bpj.2026.04.029
© 2026 The Authors. Published by Elsevier Inc. on behalf of Biophysical Society.
This is an open access article under the CC BY license (http://creativecommons.org/licenses/by/4.0/).

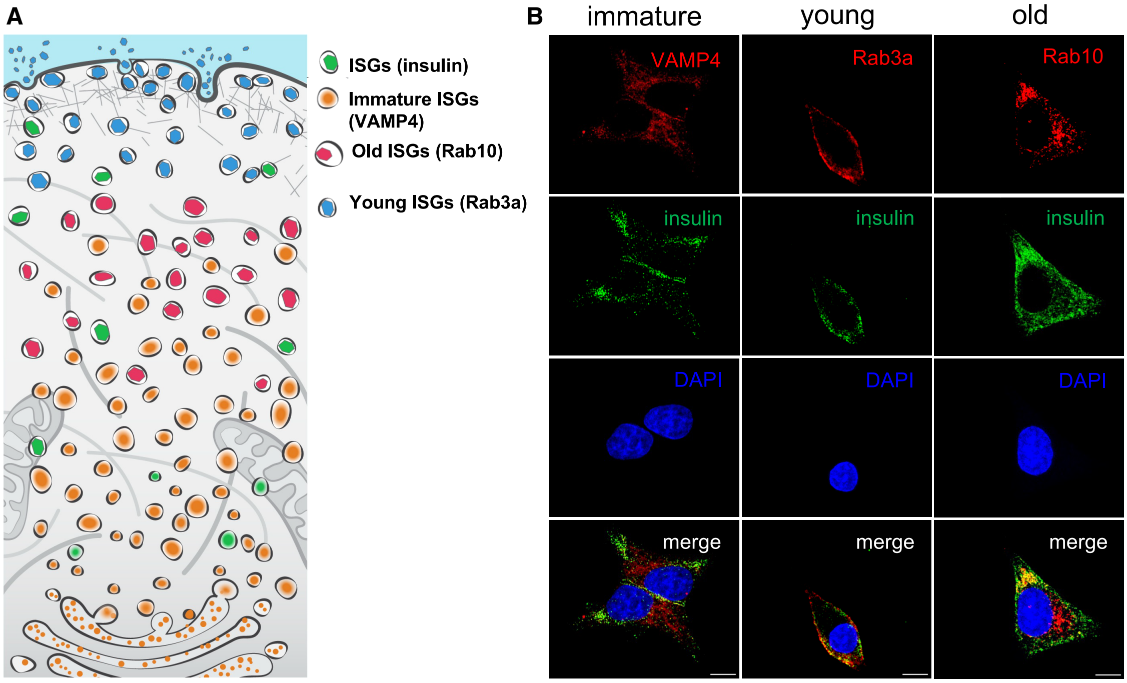

Figure 1. Insulin secretory granules form subpopulations. (A) These subpopulations have characteristic spatial distributions and functions, with immature ISGs found near the interior of the cell, young ISGs primarily found near the PM, and older ISGs found at the interior of the cell. (B) Colocalization of ISG subpopulation markers VAMP4, Rab3a, and Rab10 with insulin in INS-1E cells. Scale bar: 5 μm.

plasma membrane (PM) or mitochondria, including subpopulations such as the docked, readily releasable, or reserve pools, which are defined primarily by their distance from the PM.[15,19–22] These functional, structural, and spatial categorizations are not independent. For example, granule age correlates with mobility, distance from the PM, and release competence (Figure 1), while composition and maturity levels influence calcium sensitivity and pH.[5,6,11,18,23] For this reason, ISG subpopulations are often defined by overlapping features, complicating efforts to obtain a complete picture of biologically distinct subpopulations.

Identification of subpopulations has traditionally required *a priori* knowledge of protein markers to distinguish subtypes using fluorescence microscopy. Alternative approaches to isolating ISGs for follow-up lipidomics and proteomic analysis provide highly heterogeneous results, most likely due to differences in specific cellular conditions.[7] It has been difficult to isolate ISGs for extensive biophysical characterization, as isolated ISGs often contain contamination by proteins from many other cellular compartments, such as the endoplasmic reticulum (ER), *trans*-Golgi network, and cytoskeletal, lysosomal, and mitochondrial proteins.[7,24–31] To better understand the scope of ISG heterogeneity in the β cell, an unbiased separation method is necessary. Dielectrophoresis has been used to separate both biological and nonbiological samples, including proteins, stem cells, bacterial strains, and gold nanoparticles, among others.[32–35] Thus, in our previous work, we established direct current insulator-based dielectrophoresis (DC-iDEP) as an avenue for unbiased separation of ISG subpopulations.[36]

DC-iDEP separates particles using a nonuniform electric field based on their electrokinetic mobility ratio (EKMr). Biophysical differences between ISG subpopulations, particularly differences in radius, zeta potential or surface charge, and conductivity, affect the EKMr values of each subpopulation according to the equation

$$\mathrm{EKMr} = \frac{\mu_{EK}}{\mu_{DEP}} = \frac{\zeta_p - \zeta_m}{r^2\left(\dfrac{\sigma_p - \sigma_m}{\sigma_p + 2\sigma_m}\right)\nabla E^2}, \quad (1)$$

where μ_{EK} and μ_{DEP} refer to the electrokinetic and dielectrophoretic mobilities, respectively; ξ_p refers to the zeta potential of the particle; ξ_m refers to the zeta potential of the medium; r refers to the radius of the particle; σ_p refers to the conductivity of the particle; σ_m refers to the conductivity of the medium; and E refers to the electric field.[37] Variations in the size, protein, and lipid compositions between different ISG subpopulations are expected to influence their EKMr distributions. A sawtooth-patterned microfluidic chip is used to accumulate particles, in this case ISGs, with specific EKMr values between the triangular tips, called "gates," that have been designed to have a particular EKMr value at specific applied voltages (Figure S1).[32–35] Barekatain and Liu et al. used DC-iDEP to separate ISG subpopulations and reported significant changes in the ratios of ISG

subpopulations between unstimulated and glucose-stimulated cells.[36]

To build on this concept, we focused on ISG subpopulations defined by maturation stage, using known molecular markers, enabling us to assess the distributions of these subpopulations and their potential overlap. Immature, young, and old ISGs have been found to contain VAMP4, Rab3a, and Rab10, respectively[5,7,38] (Figure 1A). Accordingly, Rab3a-, Rab10-, and VAMP4-positive ISGs are hereafter referred to as young, old, and immature ISGs, respectively, and insulin-positive ISGs will be collectively referred to as ISGs. Immature ISGs are precursors to mature ISGs and are not typically secreted. Young, mature ISGs are more mobile and more acidic and are preferentially released over older ISGs, while older ISGs are "caged" and targeted for degradation.[5,11,18,39–42]

We also incorporate additional insulinotropic stimuli known to remodel ISGs and differentially affect ISG subpopulations, such as G-protein-coupled receptor 40 (GPR40) agonists and incretins.[15] GPR40, a free fatty acid receptor, is involved in lipid signaling and the regulation of insulin secretion. Incretins are hormones that increase glucose-stimulated insulin secretion and ISG maturation and include GLP-1 (glucagon-like peptide-1) and its analogs and GIP (glucose-dependent insulinotropic polypeptide).[16,43] These two classes of drugs likely enhance ISG maturation differently, producing unique subpopulations.[15,16] GPR40 agonists have been found to promote ISG maturation and to increase ISG diameter as the ISG approaches the periphery of the cell.[16] The incretin exendin-4 (Ex-4) has been found to promote ISG acidification and increase ISG density throughout the cell.[16,18]

In this work, we measure the distribution and overlap of ISG subpopulations associated with differences in maturity and age in the INS-1E cell line. We compare differences in this distribution as a result of orthogonal stimuli: a GPR40 agonist (TAK-875) and an incretin (Ex-4). We show that there are significant differences in the distributions of immature, young, and old ISGs and that these distributions change in characteristic ways as a result of incretin stimulation and GPR40 agonism. Understanding how unique protein signaling pathways impact ISG maturation and generation of subpopulations is a critical step for unraveling the fundamental mechanisms of secretory biology and future development of effective therapeutics.

MATERIALS AND METHODS

Cell culture

INS-1E cells (a gift from Pierre Maechler's laboratory at the University of Geneva) were cultured in 5% CO_2 at 37°C. Cells were seeded in optimized RPMI 1640 medium (AddexBio C0004-02, supplemented with 5% fetal bovine serum [FBS], 50 μM 2-mercaptoethanol, 1× penicillin-streptomycin, and 100 μg/mL streptomycin), sterile filtered through a 0.22 μm filter, and grown to 80% confluency. For each biological replicate, cells were seeded at a density of 4×10^4 cells/cm^2 in one-layer cell chambers (Avantor 734-1038). For stimulation, cells were starved in KRBH buffer containing 0 mM glucose for 30 min before stimulation in KRBH containing 25 mM glucose and either 10 nM Ex-4 or 10 μM TAK-875 for 30 min.

Colocalization of subpopulation markers with insulin

Cells were grown on ibidi 8-well high ibiTreat slides (80806-96). Cells were fixed with 4% PFA for 10 min at 4°C, then stained with a primary antibody cocktail containing guinea pig anti-insulin (1:200, BioSynth 70R-10659) and an ISG subpopulation marker (mouse anti-Rab3a, 1:100 [Synaptic Systems 107 111]; mouse anti-insulin, 1:200 [Cell Signaling Technology 8138S]; rabbit anti-Rab10, 1:100 [Cell Signaling Technology 8127S]; rabbit anti-VAMP4, 1:100 [Synaptic Systems 136 002]) in 0.5% bovine serum albumin (BSA), 0.2% saponin, and 1% FBS in Tris-buffered saline (TBS) at 4°C overnight. Excess antibody was removed by three washes with TBST (0.1% Tween-supplemented TBS) for 10 min, then cells were stained with a secondary antibody cocktail containing Alexa Fluor 488 AffiniPure goat anti-guinea pig IgG (H+L) (Jackson ImmunoResearch 106-545-003) and either Alexa Fluor 568 AffiniPure donkey anti-mouse IgG (H+L) (Jackson ImmunoResearch 715-575-150) or Alexa Fluor 568 AffiniPure goat anti-rabbit IgG (H+L) (Jackson ImmunoResearch 111-575-144) for 30 min at room temperature. Excess antibody was removed by 3 washes of 10 min each, and then cells were mounted in ProLong Glass Antifade Mountant with NucBlue Stain (Thermo Fisher Scientific P36981) and cured for 24 h before imaging.

Confocal imaging was performed using a Leica Mica microhub equipped with a 63×/1.2 NA water immersion objective. The signal was collected using 359, 499, and 650 nm excitation and 461, 520, and 668 nm emission for nuclei, insulin, and ISG subpopulation markers, respectively, by a HyD FS detector. The images were deconvoluted by LAS X, the lightning module. 15–20 planes were collected with a Z-step of 0.16 μm, and a maximum projection of the planes was produced.

ISG enrichment

Cells were harvested with 0.125% trypsin and gently washed in phosphate-buffered saline before being suspended in homogenization buffer (HB; 250 mM sucrose, 150 mM NaCl, 4 mM HEPES [pH 7.4], and 1 mM EGTA) supplemented with house-made protease inhibitor (PI) cocktail (0.5 M AEBSF, 1 mM E-64, 1.13 mM leupeptin, and 151.36 μM aprotinin). Following trypsinization, all

steps were performed at 4°C. Cells were homogenized by 20 strokes in a Dounce homogenizer (Figure 2A), then centrifuged at 1,500 × *g* for 10 min to collect cell debris. Supernatant was collected, then the pellet was resuspended in HB supplemented with PI cocktail and subjected to homogenization and centrifugation as described above. The supernatants from both spins were then centrifuged at 5,900 × *g* for 15 min to remove larger organelles. The supernatants from that spin were then pooled and centrifuged at 35,000 × *g* for 60 min to sediment ISGs. The pellet was then resuspended in 400 μL HB and layered onto a density gradient column formed by two layers of Optiprep supplemented with 1 μM EDTA (Sigma-Aldrich D1556; 3.8 mL of 35% and 0.5 mL of 14.5%) in an open-top thin-wall polypropylene tube (Beckman 326819). The density column was centrifuged in an SW55i Beckman rotor at 190,000 × *g* overnight to fractionate vesicle populations. The column-containing tube was punctured at the bottom, and 0.4 mL fractions were collected. Enzyme-linked immunosorbent assay (ELISA) was used to identify ISG-containing fractions. Similar dilutions were used for each fraction, and the manufacturer's manual was followed for ELISA (Mercodia 10-1250-01). ISG-containing fractions were then pooled, diluted with HB, and centrifuged at 40,000 × *g* for 60 min. The pelleted ISGs were resuspended in a low-conductivity buffer (LCHB; 0.3 M sucrose and 5 mM MES [pH 6.3]), which is compatible with dielectrophoresis studies. Enriched ISG samples were then confirmed to have the expected diameter by nanoparticle tracking analysis (NTA).

Western blotting

To determine the purity of the enriched ISGs, the cell pellet (post-1,500 × *g* centrifugation), the organelle pellet (post-5,900 × *g* centrifugation), and the final pellet containing enriched ISG samples were mixed with 10× NuPAGE sample reducing agent (Invitrogen NP0009) and 2× Novex Tris-glycine SDS sample buffer (Invitrogen LC2676). Samples were loaded onto Novex Tris-glycine mini protein gels (10%–20%; Invitrogen XP10205BOX) and run in a mini gel tank at 160 V for 60 min. Protein on the gel was then transferred to nitrocellulose membranes using mini iBlot 2 Transfer Stacks (Invitrogen IB23002) in an iBlot 2 dry blotting device (Invitrogen IB21001). The membrane was blocked in 5% BSA in TBST, then incubated with antibodies against marker proteins for the ER (SEC61B, Invitrogen PA3-015), endosomes (EEA1, Cell Signaling Technology 2411 S), exosomes (CD63, Novus Biologicals NB100-77913), mitochondria (Cytochrome C, Novus Biologicals NB100-56503), lysosomes (LAMP2A, Cell Signaling Technology), and ISGs (synaptotagmin 9 [syt9], Synaptic Systems 105 053) at room temperature for 5 h. Membranes were then washed three times with TBST, then incubated with alkaline phosphatase AffiniPure goat anti-mouse IgG (H+L) (Jackson ImmunoResearch

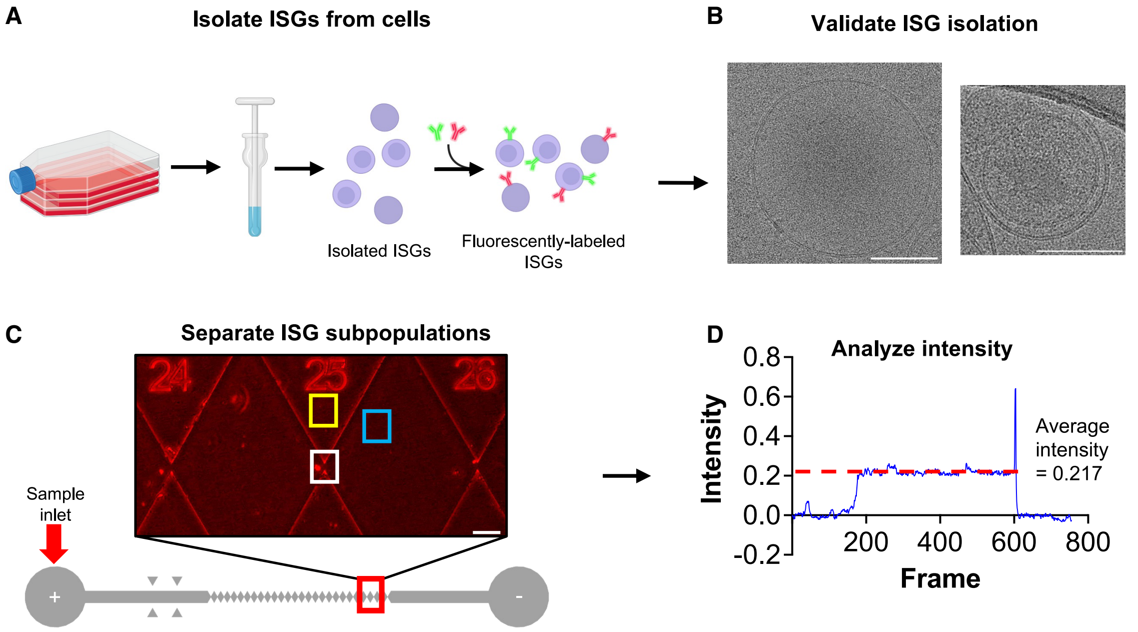

Figure 2. Workflow for separation experiments and analysis. (A) ISGs were isolated from INS-1E cells using density gradient centrifugation, then pooling insulin-rich fractions (created in BioRender). (B) ISG isolation was validated using cryo-electron microscopy and other biochemical and biophysical analyses (pictured in Figure S3). (C) Isolated ISGs were added to a sawtooth-patterned microfluidic channel and separated by applying a voltage. (D) Intensity at the gate (white box in C) is measured during a capture event and background corrected relative to the nearby channel (blue box in C) and PDMS (yellow box in C) to account for local illumination variation. A fully worked-out intensity-processing example is shown in Figure S2. Scale bars: (B) 100 nm and (C) 100 μm.

115-055-003) and alkaline phosphatase AffiniPure alpaca anti-rabbit IgG (H+L) (Jackson ImmunoResearch 611-055-215) at room temperature for 1 h. Membranes were then washed three times with PBST, and bands were visualized with 1-step NBT/BCIP substrate solution (Thermo Fisher Scientific 34042).

Visualization of ISGs by cryo-electron microscopy

All animal studies were conducted using procedures approved and conducted per Institutional Animal Care and Use Committee (IACUC) guidelines at the University of Southern California (animal use protocol #21120). Mice were 2–3 months of age for experiments. Islets from LSL-Salsa6f mice (RRID: IMSR_JAX:031968) and human islets (RRID: SCR _014387; received from the Integrated Islet Distribution Program) were isolated and dissociated into single cells.[44] Islet cells or INS-1E cells were deposited onto 200 mesh gold lacey carbon grids (Ted Pella 01894G) and allowed to adhere for 24 h in media. For stimulation conditions, cells were starved in KRBH buffer containing 0 mM glucose for 30 min before stimulation in KRBH containing 25 mM glucose and either 10 nM Ex-4 or 10 μM TAK-875 for 30 min. ISGs were isolated from INS-1E cells as described above. Excess buffer was removed from grids for 2 or 5 s for isolated ISGs or cells, respectively, at 37°C and 97% humidity, then grids were plunge frozen in liquid ethane using a Vitrobot Mark IV (Thermo Fisher Scientific). Grids were clipped using NanoSoft Autogrid rings and clips (MiTeGen M-CEM-NS-11011001).

Autogrids were screened under cryogenic conditions using a 200 kV Glacios cryo-transmission electron microscope (cryo-TEM) equipped with a Falcon 4 detector (Thermo Fisher Scientific, 13,500× or 930,00× magnification). Mouse and INS-1E cells were additionally imaged as part of the tilt series on a 300 kV Krios G3i equipped with a Gatan K3 direct detection camera (Thermo Fisher Scientific, 260,00× magnification), but only frames at a 0° tilt were analyzed in this work.

Immunolabeling of ISGs

Isolated ISGs were incubated with 1:100 each of either anti-insulin (Cell Signaling Technology #8138) and anti-VAMP4 (Synaptic Systems 136 002) or anti-Rab3a (Synaptic Systems 107 111) and anti-Rab10 (Cell Signaling #8127) overnight. ISGs were washed with LCHB, then fluorescently labeled by incubation with 1:100 each of an Alexa 488-conjugated secondary antibody (Jackson ImmunoResearch 115-545-146) and an Alexa 568-conjugated secondary antibody (Jackson ImmunoResearch 111-575-144) for 2 h. Fluorescently labeled ISGs were then washed once with LCHB to remove excess antibody and finally resuspended in LCHB.

Device fabrication

The microfluidic device was fabricated and designed as described in previous publications.[36,45] There are 27 gates (paired triangle tips) ranging in size from 25 to 73 μm, with wider gates closer to the inlet. The width of the gates decreases approximately 5 μm after every three repeats. DC was applied to the inlet and outlet, with potentials of 900, 1,200, and 1,500 V tested.

Separation of ISG subpopulations by DC-iDEP

The separation channel was pretreated with 4% BSA for 15 min, then washed with LCHB. 10–15 μL of an ISG sample was added to the inlet, and the volume in the channel was maintained at the outlet by adding LCHB to prevent pressure-driven flow. DC was applied at 900, 1,200, and 1,500 V using a 3,000D high-voltage sequencer (LabSmith HVS448), and particles were allowed to move through the channel according to their EKMr values (Figure 2C).

Separation experiments were imaged on a Leica DM IL LED Fluo inverted laboratory microscope equipped with an S80/0.30 condenser and a 5×/0.12 NA objective. Videos were recorded using a Leica Flexacam C1 camera at 1,080-pixel resolution and 30 frames per second. A Leica EL6000 external light source was used with a 3-position fluorescence slider for fluorescence excitation.

Normalization

Biological replicates were defined as independent ISG preparations derived from separate cell culture flasks. Technical replicates refer to independent experiments performed on aliquots from the same bulk ISG preparation. Intensity values were measured for the duration of each recording in three locations per gate: (1) the gate, (2) the PDMS near the gate, and (3) an empty location in the channel using Fiji (Figure 2C).[46] A capture event is defined as the presence of one or more fluorescent particles visibly retained at the gate for at least 30 frames (1 s, Video S1). Intensity values were averaged during a capture event. Gates with no capture events were given an intensity value of 0. To correct for spatial variation in illumination, the gate intensity when empty was subtracted from the intensity during a capture event and normalized to the average of the PDMS and channel intensities (Figure S2A):

$$I_{corrected} = \frac{I_{gate,capture} - I_{gate,empty}}{\frac{I_{channel} + I_{PDMS}}{2}}. \quad (2)$$

Gates with identical EKMr values were averaged.

To account for differences in fluorophore brightness and overall sample concentration between biological replicates,

each dataset was independently scaled to a 0–1 range based on the minimum and maximum intensities (I_{min} and I_{max}, respectively) (Figure S2B). For each gate, the scaled intensity was calculated as

$$I_{scaled} = \frac{I_{corrected} - I_{min}}{I_{max} - I_{min}}. \quad (3)$$

These final normalized values were plotted against their corresponding EKMr values (Figure S2C).

Selection of applied voltage

We used three applied voltages to separate ISG subpopulations by DC-iDEP: 900, 1,200, and 1,500 V (Figure S3). Each voltage affects the environment of the microfluidic channel differently, thereby changing the EKMr of each gate in the channel. The EKMr distributions are different at each voltage due to binning, as the microfluidic channel has gates with discrete rather than continuous EKMr values. With this channel design, lower voltages have greater separation at lower EKMr values, whereas higher voltages allow for better separation of particles with higher EKMr values. Due to this binning, particles that are separated at lower voltages are captured at the same gate at higher voltages. Ultimately, we decided to focus on the 1,500 V datasets due to the broader EKMr range (Figure S4). Given the strong inverse relationship between EKMr and radius, particles with higher EKMr values are predicted to correspond to smaller ISGs.

RESULTS

Localization of ISG subpopulations in INS-1E cells

We chose to focus on ISG subpopulations that reflect key stages of maturation and fluorescently labeled these subpopulations based on previously reported membrane markers. Upon fluorescently labeling ISG subpopulation markers for immature, young, and old ISGs, these subpopulations localized to distinct regions of the cell, consistent with prior studies (Figure 1B).[5,11,18,47,48] VAMP4 (immature ISGs) did not colocalize with insulin because proinsulin had not been processed to insulin yet and was primarily found far from the PM. Rab3a (young ISGs) was largely present at the periphery of the cell and had high colocalization with insulin, suggesting these ISGs are mature and ready for secretion.[13,19,22] Rab10 (old ISGs) was mostly present in the interior of the cell, consistent with findings of old ISGs being caged in the interior of the cell.[42,49]

Analysis of ISGs post-isolation

Next, we isolated ISGs from INS-1E cells. Isolated fractions from density gradient centrifugation were analyzed by insulin ELISA. Fractions 8–10 were identified as ISG-containing fractions (Figure S5A) and were pooled and analyzed using a variety of biochemical and biophysical analyses (Figures 2B and S5). Western blotting showed that ISG (syt9), lysosomal (LAMP2A), and exosomal (CD63) markers were enriched in pooled ISG samples. The presence of the exosomal marker CD63 in isolated ISGs is consistent with the finding of exosomes inside dense core vesicles but may also indicate the presence of contaminants.[50] LAMP2A has also been shown to colocalize with ISGs, but its presence may be indicative of potential contamination.[30] Fluorescently labeling ISG subpopulation markers prevented contaminating particles from being analyzed. ER, endosome, and mitochondrial markers were found in cell and organelle lysates but not enriched ISG samples (Figure S5B). NTA and cryo-electron microscopy (cryo-EM) both confirmed the enriched ISG samples contained particles of the expected size (diameter: 200–500 nm; Figures 2B and S5C).[19,51]

Comparison of ISG subpopulation distributions in unstimulated cells

We then fluorescently labeled ISGs using ISG subpopulation-specific markers and separated them by DC-iDEP. Separation of ISG subpopulations by DC-iDEP revealed different EKMr distributions within them (Figure 3A). Insulin was used as an overall ISG marker, and the unweighted sum of the independently normalized VAMP4, Rab3a, and Rab10 distributions largely correlates with that of insulin (Figure S6), indicating that the three subpopulations encompass most ISG subpopulation states. The immature ISG marker VAMP4 exhibited a heterogeneous distribution, characterized by low-EKMr ISGs.[7] Independent biological replicates exhibited similar EKMr profiles with expected variability across bins (Figure S7). Rab3a, a young ISG marker, had a bimodal EKMr distribution at mid-range values, while the old ISG marker Rab10 displayed ISGs at high EKMr values (Figure 3A).[5] Differences between subpopulation markers were analyzed via two-way ANOVA with Bonferroni post hoc correction (Table S1). Significant differences were found between young and old ISGs (9.30×10^9 V/m^2, $p = 0.0495$; 1.45×10^{10} V/m^2, $p = 0.0461$; and 2.23×10^{10} V/m^2, $p = 0.0178$), immature ISGs and insulin (4.10×10^9 V/m^2, $p = 0.0157$), and immature and old ISGs (1.45×10^{10} V/m^2, $p = 0.0489$) (Figures 3B and 3C). These differences indicate that each population has distinct distribution patterns due to the biophysical differences in ISGs, with overlap between distributions likely indicating overlapping subpopulations. This overlap between subpopulations is likely especially present within young ISGs, as this pool probably contains ISGs with a range of maturity levels.

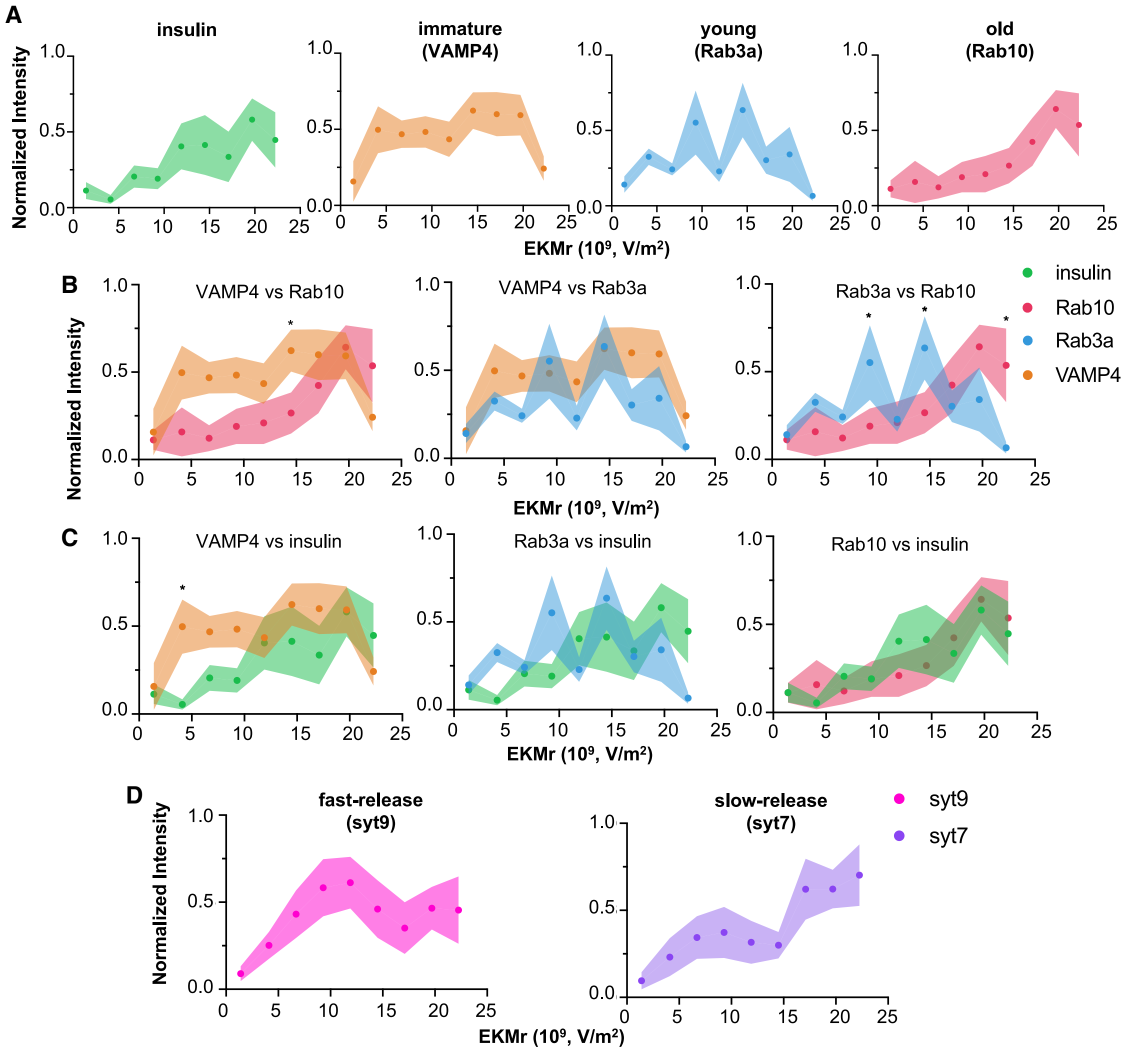

Figure 3. ISG subpopulations associated with different maturation stages have characteristic EKMr distributions. (A) EKMr distributions of ISGs in unstimulated cells ($n = 3$–4 biologically independent experiments). (B) Comparisons of the distributions of different ISG subpopulations. (C) Comparisons of the distributions of each subpopulation with the distribution of insulin. (D) EKMr distributions of two additional marker-defined ISG pools, synaptotagmin 9 (syt9; fast release) and synaptotagmin 7 (syt7; slow release), shown for comparison to previously characterized functional subpopulations. Values are the mean ± SEM (*$p \leq 0.05$ using ANOVA with Bonferroni post hoc multiple comparison correction).

Effect of insulinotropic drugs on ISG subpopulations

To better understand how insulinotropic signals impact ISG remodeling or shifts in distinct subpopulations, we compared EKMr distributions of ISGs in unstimulated cells with those in cells stimulated by either TAK-875 or Ex-4. Both stimuli had significant effects on the EKMr distribution of isolated ISGs when analyzed via two-way ANOVA with Bonferroni post hoc correction, with TAK-875 producing larger shifts than Ex-4 across all applied voltages (Figure S8).

TAK-875 significantly decreased immature ISGs at several EKMr values, while Ex-4 did not produce significant changes at individual EKMr values. Specifically, immature ISGs were decreased by TAK-875 (4.10×10^9 V/m², $p = 0.0264$; 6.70×10^9 V/m², $p = 0.0345$; and 1.45×10^{10} V/m², $p = 0.0243$; Figure 4A). Both TAK-875 and Ex-4 stimulation affected the shape of the EKMr distribution of immature ISGs. When stimulated by either TAK-875 or Ex-4, there was a smaller proportion of ISGs containing VAMP4 at lower EKMr values than in the unstimulated condition (Figure 4A). In contrast to the significantly decreased intensity of immature ISGs at 1.45×10^{10} V/m² in response to TAK-875, Ex-4 stimulation led to a single peak in the EKMr distribution at the same EKMr value (Figure 4A), potentially revealing a condition-dependent maturation pathway producing mid-sized ISGs shared by both TAK-875 and Ex-4 stimulation.

Both TAK-875 and Ex-4 caused significant changes in the EKMr distribution of young ISGs at 1,500 V (Figure 4A). TAK-875 induced a significant decrease in young ISGs at

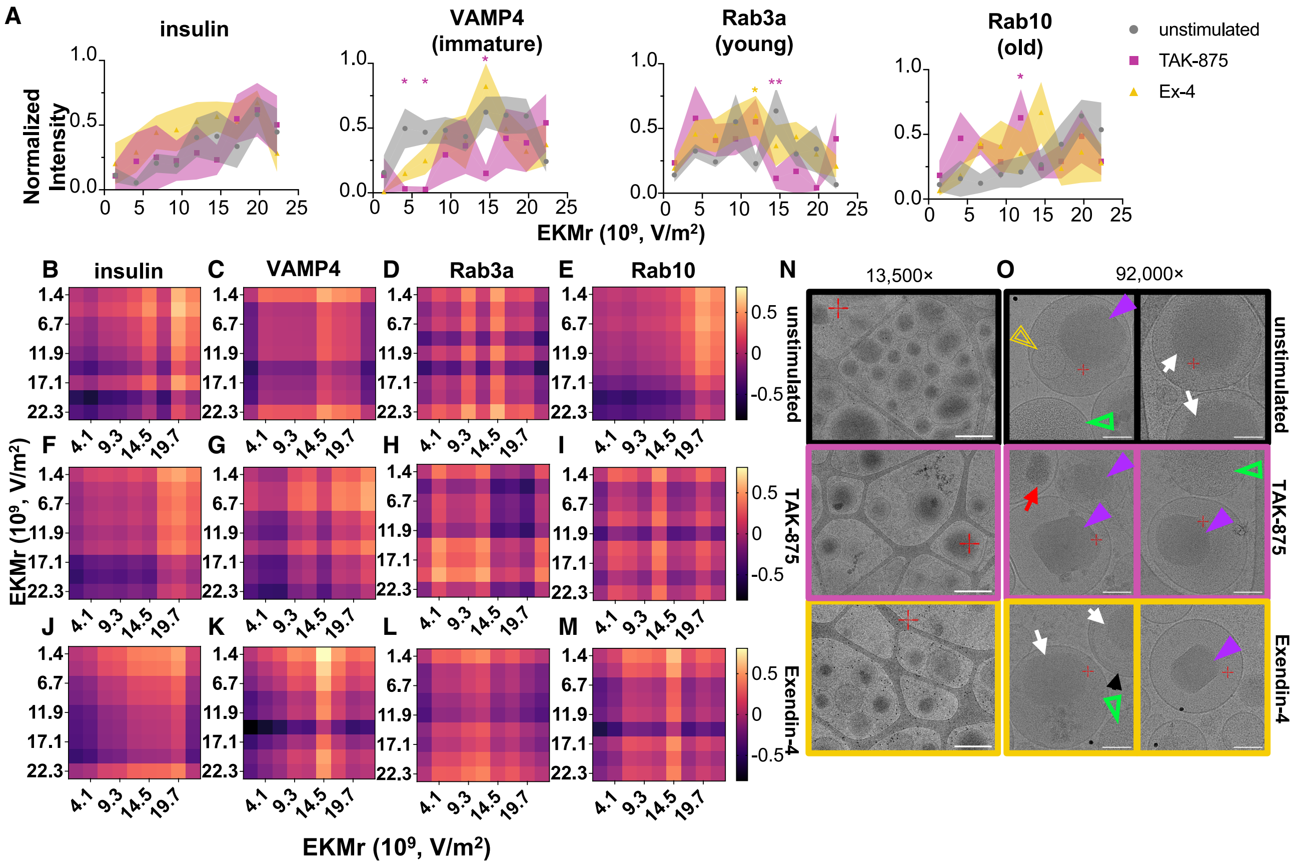

Figure 4. ISG subpopulations undergo remodeling in response to insulinotropic stimulation. (A) EKMr distributions of ISGs isolated from unstimulated, TAK-875-stimulated ($n = 2$ biologically independent experiments), and Ex-4-stimulated ($n = 2$–3 biologically independent experiments) cells. (B–M) Heatmaps displaying differences in average intensities between each pair of EKMr values within ISG subpopulation markers and (B–E) unstimulated, (F–I) TAK-875-stimulated, and (J–M) Ex-4-stimulated conditions. (N and O) Cryo-electron micrographs of ISGs inside unstimulated (*top*), Ex-4-stimulated (*middle*), and TAK-875-stimulated (*bottom*) mouse primary β cells at (N) 13,500× magnification, pixel size 2.06 nm, and (O) 92,000× magnification, pixel size 0.15 nm. (O) Symbols depict vesicles inside ISGs (*yellow*, *double-lined arrowheads*), crystalline cores (*purple arrowheads*), condensing cores (*white arrows*), immature ISGs (*green*, *empty arrowheads*), aggregates (*red arrow*), and fibrils (*black arrow*). Values are the mean ± SEM (*$p \leq 0.05$ and **$p \leq 0.01$ using ANOVA with Bonferroni post hoc multiple comparison correction; comparisons were made between stimulation and unstimulated conditions; symbols are color coded according to condition). Scale bars: (N) 500 nm and (O) 100 nm.

1.45×10^{10} V/m^2 ($p = 0.00960$), while Ex-4 increased the abundance of ISGs at 1.19×10^{10} V/m^2 ($p = 0.0441$). TAK-875 and Ex-4 both eliminated the bimodal distribution of young ISGs observed in unstimulated cells, with TAK-875 favoring low EKMr values, consistent with a shift toward larger ISGs, and Ex-4 producing a more heterogeneous distribution (Figure 4A).[16]

Old ISGs also exhibited significant remodeling in response to stimulation. In the TAK-875 condition, old ISGs increased significantly at 1,500 V (1.19×10^{10} V/m^2, $p = 0.0417$; Figure 4A), potentially corresponding to mid-sized ISGs. Notably, both TAK-875 and Ex-4 had significant effects on old ISGs at multiple EKMr values at both 900 and 1,500 V (Table S2; Figure S8D). Both TAK-875 and Ex-4 increased the proportion of old ISGs with lower EKMr values than in the unstimulated condition, with lower-EKMr peaks in the TAK-875 condition than in the Ex-4 condition.

To understand how multiple subpopulations may be similarly affected by the same condition, which might not be obvious in the original plots, we visualized relative EKMr shifts using heatmaps generated from normalized intensity differences within a single subpopulation and condition (Figures 4B–4M). These heatmaps allowed for the identification of other trends in EKMr distribution in response to stimulation. For example, in response to Ex-4, a similar peak developed at 1.45×10^{10} V/m^2 in immature and old ISGs (Figure 4A), displayed in the heatmap as a dark horizontal or light vertical band (Figures 4K and 4M). Similarly, VAMP4 heatmaps show vertical bands at 1.45×10^{10} V/m^2, indicating opposite effects on the relative amount of ISGs at the same EKMr value in response to TAK-875 and Ex-4 (Figures 4G and 4K). There were few significant differences between TAK-875 and Ex-4 stimulation, with Ex-4-stimulated cells having a significantly higher ratio of ISGs at 1.45×10^{10} V/m^2

($p = 0.00410$), but the shapes of each were often different (Figure S9; Table S3).

As antibody binding could influence zeta potential and affect EKMr distributions, we examined whether the identity of a second antibody altered the EKMr distribution associated with young ISGs. ISGs labeled with Rab3a in combination with either VAMP4 or syt9 exhibited qualitatively similar EKMr distributions (Figure S10). No significant differences were detected between each dual-labeling scheme across EKMr values.

To visualize physical differences in ISGs under these different conditions, we imaged primary mouse β cells using cryo-EM. There were observable differences in the appearance of ISGs in unstimulated cells compared with TAK-875- or Ex-4-stimulated cells. Unstimulated cells contained many ISGs with various levels of insulin condensation, as well as some ISGs containing exosome-like structures.[50] As expected, based on soft X-ray tomography (SXT) experiments, the number of ISGs present near the cell periphery in TAK-875 and Ex-4 conditions was lower than in unstimulated cells (Figure 4N).[16] TAK-875 stimulation caused higher levels of crystallization and more ordered crystals. In this condition, we also observed the presence of dark, unidentified aggregates attached to the cores of some ISGs that likely contributed to our previous observation of denser ISGs (Figure 4O).[16] Ex-4 stimulation led to increased crystallization, denser cores, and some ISGs with a smaller crystal-to-vesicle ratio than in the unstimulated condition (Figures 4N and 4O).

DISCUSSION

Subpopulations of ISGs have varying sizes and compositions, but the extent of these differences is unknown.[5,6,15,16,18] In this work, our goal was to further develop separation methods and provide a more comprehensive analysis of the biophysical features of ISG subpopulations.[36] In particular, we wanted to increase the interpretability of DC-iDEP separations by identifying the distribution of previously identified ISG subpopulations using Rab3a, Rab10, and VAMP4 antibodies to label young, old, and immature ISGs, as well as an insulin antibody to label all maturing ISGs.[5,7] In this work, we found that these ISG subpopulations had characteristic distributions in unstimulated cells, indicating that known ISG pools exhibit distinct yet overlapping biophysical distributions (Figure 3A).

Interpreting the size of ISGs and EKMr distributions

The traditional view in the field is that immature ISGs tend to be larger and less dense than mature ISGs. EKMr values vary inversely with radius (Equation 1).[16,52,53] This correlates with immature ISGs displaying higher ratios of ISGs at lower EKMr values, whereas young and old ISGs displayed distribution peaks at higher EKMr values. Insulin-positive ISGs exhibited two peaks at high EKMr values, likely reflecting enrichment of smaller, more mature ISGs (Figure 3A). These trends suggest that differences in ISG size contribute substantially to the observed EKMr distributions, although size alone cannot fully account for the observed heterogeneity.

Zeta potential and conductivity of ISGs and EKMr distributions

In addition to size, differences in zeta potential and conductivity influence the EKMr value of a particle (Equation 1). Previous work has shown that young ISGs have lower zeta potentials and higher rigidities due to specific lipid compositions than older ISGs.[5] These characteristics complement the distribution peaks at lower EKMr values of young ISGs than old ISGs in this work, with immature VAMP4 ISGs corresponding to low-EKMr values (Figure 3A). Intravesicular pH may further contribute to EKMr differences, as mature ISGs exhibit lower luminal pH, which could increase conductivity and thus contribute to shifts in EKMr distributions.[18,54] Further biophysical characterization of the ISG subpopulations is necessary to completely understand which protein and lipid species are most influential in subpopulation formation.

Heterogeneity within known subpopulations

In addition to the overall distribution trends, we observed substantial heterogeneity in the form of broad or multimodal EKMr distributions of ISGs within each subpopulation we studied. This indicates that there is appreciable heterogeneity in the biophysical makeup of these ISG subpopulations. Thus, each of the known subpopulations contains a collection of physically distinct ISGs. Insulin-labeled ISGs exhibited two major peaks that overlapped with the higher-EKMr peaks seen in young and old subpopulations, consistent with the insulin antibody labeling a broad range of maturing granules. We observed the most heterogeneity in immature and young ISGs, which may be due to activation of multiple maturation pathways giving rise to distinct subtypes over the lifetime of an ISG. VAMP4-positive ISGs had a broad distribution across the EKMr range tested, with a higher ratio of low-EKMr ISGs than other subpopulations (Figure 3A). The bimodal distribution of young ISGs potentially corresponds to the presence of young ISGs with a crystalline core (1.45×10^{10} V/m^2) and young ISGs containing only condensed insulin (9.30×10^{9} V/m^2), as the subset of ISGs positive for Rab3a likely contains ISGs at various stages of maturity.[5] The heterogeneity observed within immature, young, and old subpopulations may be due to the presence of other previously described ISG pools, such as syt7 or syt9 subpopulations (Figure 3D).[6] These

subpopulations exhibit variations in their size, protein, and lipid compositions, which would affect their EKMr distribution, and are likely to exist at different maturation and age stages. In particular, the smaller radius of syt7 ISGs likely increases the average EKMr of these granules, but this effect is somewhat attenuated by the increased cholesterol increasing the rigidity of the membrane of syt7 granules (Figure 3D).[6] Additional experiments probing these subpopulations and their changes in response to various conditions would provide valuable insight into ISG maturation.

Impact of ISG maturation stimuli on ISG subpopulations

To better understand how different cellular pathways impact the generation of biophysically and functionally distinct ISG subtypes, we examined the effects of lipid signaling and incretin signaling on immature, young, and old ISGs. Two drugs that enhance ISG maturation through distinct signaling pathways, TAK-875 and Ex-4, have previously been reported to upregulate biophysically distinct ISG subpopulations.[15,16,18] TAK-875 activates lipid signaling, and TAK-875 stimulation results in an increase in larger, denser ISGs that are often closer to the PM and distinct aggregate formations within the lumen (Figure 4O).[15,16] In contrast, Ex-4 stimulation causes more condensed crystalline cores throughout the cell (Figures 4N and 4O).[15,16]

Despite these differences, stimulation by TAK-875 or Ex-4 did not result in significant changes in the distribution of insulin-labeled ISGs, although Ex-4-treated cells exhibited a modest increase in the ratio of low-EKMr to high-EKMr insulin-labeled ISGs (Figure 4A). The unweighted sum of the EKMr distributions of these subpopulations does not overlay with the distribution of insulin as cleanly as in the unstimulated condition (Figure S6), suggesting that stimulation alters the relative abundance of ISG subpopulations rather than uniformly shifting all ISGs.

We saw a decrease in the subset of ISGs containing VAMP4 with low EKMr values in response to TAK-875 (Figure 4A), consistent with an increase in mature ISGs. Stimulation with TAK-875 also resulted in an increased proportion of low-EKMr young and old ISGs, accompanied by a reduction in immature ISGs compared with the unstimulated condition. This may indicate a rise in the radius of mature ISGs in response to TAK-875, consistent with previous studies. Stimulation with Ex-4 results in increased ISG maturation, decreased ISG diameter, and decreased ISG pH.[15,16,18] We observed a decrease in low-EKMr VAMP4 ISGs, as well as a significant increase in young ISGs at 1.19×10^{10} V/m^2, in response to Ex-4 (Figure 4A), indicating a decrease in immature ISGs and likely an increase in smaller, mature ISGs containing crystalline insulin.

Interestingly, both TAK-875 and Ex-4 stimulation were associated with modest but reproducible decreases in the abundance of old ISGs at 1.97×10^{10} V/m^2. Although some changes in old ISG distributions could arise indirectly from the preferential secretion of young ISGs, β cells are known to retain and cage older ISGs.[11,18] Therefore, the observed shifts in old ISG distributions in response to TAK-875 and Ex-4 likely reflect some transformation or degradation of old ISGs rather than secretion alone. These decreases, while slight, may indicate remodeling not only of immature and newly formed ISGs but also of old ISGs.

The more pronounced effects of TAK-875 stimulation compared to Ex-4 stimulation may be due to lipid remodeling of the ISG membrane, which would be expected to alter the size and surface charges of ISGs.[16] As the field learns more about inter-organelle contacts and communication, we will be able to piece together how these physical changes occur and specific lipids and proteins that are involved in regulating ISG maturation. Given that different cellular signals impact ISG biophysical characteristics, it is clear that there are multiple pathways for maturation and a diverse range of ISGs the cell can generate.

Future exploration of the effect of diseased states may improve our understanding of the functional implications of these changes, as certain subpopulations are known to be associated with either type 1 or type 2 diabetes.[6] Identifying any overlap between the subpopulations described here and disease-related pools may enable a better understanding of these conditions and the development of future treatments. More broadly, this method may also prove useful in studying heterogeneity in other organelles or cells.[55–61]

Limitations of the study

A key limitation of separation by DC-iDEP is that ISGs must be removed from the cellular environment, which prevents a direct correlation between an ISG's EKMr value and its native surroundings. Additionally, it can be challenging to isolate all ISGs for analysis. Immature ISGs lack the dense core that mature ISGs have and are found in different fractions than mature ISGs when isolated using traditional methods such as density gradient columns.[7] To address this challenge, future development of the separation channel to allow for the removal of separated subpopulations would enable the identification of more specific subpopulation markers. Labeling and imaging these specific markers *in cellulo* would assist in the interpretation of the identities of the separated subpopulations.

Additionally, modifying the channel to increase the resolution of mid- to high-EKMr values may improve the separation of ISG subpopulations. Because DC-iDEP provides higher-sensitivity separations, further development as a preparative tool has powerful potential to methodically explore biophysical heterogeneity of ISGs, viruses, or other biologically complex particles.

INS-1E cells were used as a model of β cells in this study for their ease of use and scalability, but primary mouse or human β cells may provide more insight into the heterogeneity of ISGs during disease progression. Primary β cells appear to contain more crystalline insulin within their ISGs (Figure S11), which would likely affect the EKMr distribution. They also contain other components that might affect the EKMr distribution, such as fibrils, aggregates, and vesicles (Figures 4N and 4O).

The markers we used likely label several types of ISGs within the major subpopulations studied. Additionally, the antibodies themselves may modestly alter the EKMr of the ISGs they label. Because unlabeled ISGs cannot be analyzed in this system, we cannot directly quantify antibody-induced shifts. Comparisons using dual-labeling schemes suggest that relative differences between ISG subpopulations are robust to antibody choice (Figure S10). Each of these factors poses challenges for data interpretation, which may be made simpler by future technological advances. Despite these complications, these results provide new insights into the heterogeneity of ISGs and a basis for further use of DC-iDEP to explore biological particles. Together, these findings establish DC-iDEP as a sensitive platform for resolving biophysical heterogeneity within organelle populations and provide a framework for linking physical properties to functional states.

DATA AVAILABILITY

Data will be shared upon a reasonable request made to the corresponding author, Kate L. White (katewhit@usc.edu).

ACKNOWLEDGMENTS

This material is based on work supported by the National Science Foundation Graduate Research Fellowship Program under grant no. DGE-1842487. Any opinions, findings, and conclusions or recommendations expressed in this material are those of the author(s) and do not necessarily reflect the views of the National Science Foundation. Funding from the National Institute of General Medical Sciences of the National Institutes of Health (NIH), under award number R35GM154893, and the University of Southern California (USC) Bridge Institute at USC provided financial support for this work. Thank you to Kevin Chang and Janielle Cuala for providing mouse islets for cryo-EM experiments and Yekaterina Kadyshevskaya and Riva Verma for illustration assistance. Thank you to Alex Ramirez for assisting in setting up the DEP device at USC. Human pancreatic islets were provided by the NIDDK-funded Integrated Islet Distribution Program (IIDP) (RRID: SCR_014387) at City of Hope, NIH grant no. U24DK098085. Images presented in this article were acquired at the Core Center of Excellence in Nano Imaging at USC.

AUTHOR CONTRIBUTIONS

Conceptualization, A.A. and K.L.W.; methodology, A.A., T.K., M.A.H., and K.L.W.; investigation, A.A., T.K., and A.D.; writing – original draft, A.A. and K.L.W.; writing – review & editing, A.A., K.L.W., T.K., A.D., and M.A.H.; funding acquisition, K.L.W.; resources, K.L.W. and M.A.H.; supervision, K.L.W. and M.A.H.

DECLARATION OF INTERESTS

M.A.H. declares a conflict of interest with Hayes Diagnostics, Inc., where he serves as COB, CEO, and CSO.

SUPPLEMENTAL INFORMATION

Supplemental information can be found online at https://doi.org/10.1016/j.bpj.2026.04.029.

REFERENCES

1. Petersen, M. C., and G. I. Shulman. 2018. Mechanisms of Insulin Action and Insulin Resistance. *Physiol. Rev.* 98:2133–2223. https://doi.org/10.1152/physrev.00063.2017.
2. Hou, J. C., L. Min, and J. E. Pessin. 2009. Insulin granule biogenesis, trafficking and exocytosis. *Vitam. Horm.* 80:473–506. https://doi.org/10.1016/S0083-6729(08)00616-X.
3. Arvan, P., and D. Castle. 1998. Sorting and storage during secretory granule biogenesis: looking backward and looking forward. *Biochem. J.* 332:593–610. https://doi.org/10.1042/bj3320593.
4. Dodson, G., and D. Steiner. 1998. The role of assembly in insulin's biosynthesis. *Curr. Opin. Struct. Biol.* 8:189–194. https://doi.org/10.1016/s0959-440x(98)80037-7.
5. Neukam, M., P. Sala, ..., M. Solimena. 2024. Purification of time-resolved insulin granules reveals proteomic and lipidomic changes during granule aging. *Cell Rep.* 43:113836. https://doi.org/10.1016/j.celrep.2024.113836.
6. Kreutzberger, A. J. B., V. Kiessling, ..., L. K. Tamm. 2020. Distinct insulin granule subpopulations implicated in the secretory pathology of diabetes types 1 and 2. *eLife.* 9:e62506. https://doi.org/10.7554/eLife.62506.
7. Norris, N., B. Yau, and M. A. Kebede. 2021. Isolation and Proteomics of the Insulin Secretory Granule. *Metabolites.* 11:288. https://doi.org/10.3390/metabo11050288.
8. Vasiljević, J., J. M. Torkko, ..., M. Solimena. 2020. The making of insulin in health and disease. *Diabetologia.* 63:1981–1989. https://doi.org/10.1007/s00125-020-05192-7.
9. DJ, M., and B. EJ. 2021. Chapter 3: Global Picture. *In* IDF Diabetes Atlas International Diabetes Federation.
10. Barg, S., L. Eliasson, ..., P. Rorsman. 2002. A Subset of 50 Secretory Granules in Close Contact With L-Type Ca2 Channels Accounts for First-Phase Insulin Secretion in Mouse β-Cells. *Diabetes.* 51. https://doi.org/10.2337/diabetes.51.2007.s74.
11. Yau, B., L. Hays, ..., M. A. Kebede. 2020. A fluorescent timer reporter enables sorting of insulin secretory granules by age. *J. Biol. Chem.* 295:8901–8911. https://doi.org/10.1074/jbc.RA120.012432.
12. Yi, H., D. Gong, ..., N. F. Scherer. 2024. Distinct Sub- to Superdiffuse Insulin Granule Transport Behaviors in beta-Cells Are Strongly Affected by Granule Age. *J. Phys. Chem. B.* 128:6246–6256. https://doi.org/10.1021/acs.jpcb.4c01403.
13. Izumi, T. 2023. Multiple pathways and independent functional pools in insulin granule exocytosis. *Genes Cells.* 28:471–481. https://doi.org/10.1111/gtc.13029.
14. Kasai, K., T. Fujita, ..., T. Izumi. 2008. Docking is not a prerequisite but a temporal constraint for fusion of secretory granules. *Traffic.* 9:1191–1203. https://doi.org/10.1111/j.1600-0854.2008.00744.x.
15. Chang, K., A. Deshmukh, ..., K. L. White. 2026. Classifying biophysical subpopulations of insulin secretory granules using quantitative whole cell structure analysis. *Structure*. https://doi.org/10.1016/j.str.2026.03.005.
16. Deshmukh, A., K. Chang, ..., K. L. White. 2025. Secretory stimuli distinctly regulate insulin secretory granule maturation through structural remodeling. *Structure.* 33:1831–1843.e4. https://doi.org/10.1016/j.str.2025.07.022.

17. Hosaka, M., T. Watanabe, …, T. Izumi. 2007. A subset of p23 localized on secretory granules in pancreatic beta-cells. *J. Histochem. Cytochem.* 55:235–245. https://doi.org/10.1369/jhc.6A7093.2006.

18. Lin, W., K. Tseng, …, K. L. White. 2024. Decoding Insulin Secretory Granule Maturation Using Genetically Encoded pH Sensors. *ACS Sens.* 9:6032–6039. https://doi.org/10.1021/acssensors.4c01885.

19. Olofsson, C. S., S. O. Göpel, …, L. Eliasson. 2002. Fast insulin secretion reflects exocytosis of docked granules in mouse pancreatic B-cells. *Pflügers Archiv.* 444:43–51. https://doi.org/10.1007/s00424-002-0781-5.

20. Olofsson, C. S., A. Salehi, …, P. Rorsman. 2004. Palmitate increases L-type Ca2+ currents and the size of the readily releasable granule pool in mouse pancreatic β-cells. *J. Physiol.* 557:935–948. https://doi.org/10.1113/jphysiol.2004.066258.

21. Pottekat, A., S. Becker, …, W. E. Balch. 2013. Insulin Biosynthetic Interaction Network Component, TMEM24, Facilitates Insulin Reserve Pool Release. *Cell Rep.* 4:921–930. https://doi.org/10.1016/j.celrep.2013.07.050.

22. Daniel, S., M. Noda, …, G. W. Sharp. 1999. Identification of the docked granule pool responsible for the first phase of glucose-stimulated insulin secretion. *Diabetes.* 48:1686–1690. https://doi.org/10.2337/diabetes.48.9.1686.

23. Hoboth, P., A. Müller, …, M. Solimena. 2015. Aged insulin granules display reduced microtubule-dependent mobility and are disposed within actin-positive multigranular bodies. *Proc. Natl. Acad. Sci. USA.* 112:E667–E676. https://doi.org/10.1073/pnas.1409542112.

24. Tooze, S. A., T. Flatmark, …, W. B. Huttner. 1991. Characterization of the immature secretory granule, an intermediate in granule biogenesis. *J. Cell Biol.* 115:1491–1503. https://doi.org/10.1083/jcb.115.6.1491.

25. Li, M., W. Du, …, J. Hou. 2018. Proteomic analysis of insulin secretory granules in INS-1 cells by protein correlation profiling. *Biophys. Rep.* 4:329–338. https://doi.org/10.1007/s41048-018-0061-3.

26. MacDonald, M. J., L. Ade, …, S. W. Stoker. 2015. Characterization of phospholipids in insulin secretory granules and mitochondria in pancreatic beta cells and their changes with glucose stimulation. *J. Biol. Chem.* 290:11075–11092. https://doi.org/10.1074/jbc.M114.628420.

27. Hickey, A. J. R., J. W. I. Bradley, …, G. J. S. Cooper. 2009. Proteins Associated with Immunopurified Granules from a Model Pancreatic Islet β-Cell System: Proteomic Snapshot of an Endocrine Secretory Granule. *J. Proteome Res.* 8:178–186. https://doi.org/10.1021/pr800675k.

28. Guest, P. C. 2017. Multiplex Sequential Immunoprecipitation of Insulin Secretory Granule Proteins from Radiolabeled Pancreatic Islets. *Methods Mol. Biol.* 1546:177–185. https://doi.org/10.1007/978-1-4939-6730-8_14.

29. Albrethsen, J., J. P. Goetze, and A. H. Johnsen. 2015. Mining the Granule Proteome: A Potential Source of Endocrine Biomarkers. *Biomark. Med.* 9:259–265. https://doi.org/10.2217/bmm.14.107.

30. Brunner, Y., Y. Couté, …, J. C. Sanchez. 2007. Proteomics analysis of insulin secretory granules. *Mol. Cell. Proteomics.* 6:1007–1017. https://doi.org/10.1074/mcp.M600443-MCP200.

31. Schvartz, D., Y. Brunner, …, J. C. Sanchez. 2012. Improved characterization of the insulin secretory granule proteomes. *J. Proteomics.* 75:4620–4631. https://doi.org/10.1016/j.jprot.2012.04.023.

32. Zhu, F., B. L. Nannenga, and M. A. Hayes. 2019. Electrophoretic exclusion microscale sample preparation for cryo-EM structural determination of proteins. *Biomicrofluidics.* 13:054112. https://doi.org/10.1063/1.5124311.

33. Liu, Y., A. Jiang, …, M. A. Hayes. 2019. Identification of neural stem and progenitor cell subpopulations using DC insulator-based dielectrophoresis. *Analyst.* 144:4066–4072. https://doi.org/10.1039/c9an00456d.

34. Liu, Y., and M. A. Hayes. 2020. Differential Biophysical Behaviors of Closely Related Strains of Salmonella. *Front. Microbiol.* 11:302–2020. (Original Research). https://doi.org/10.3389/fmicb.2020.00302.

35. Ramirez, A. J., A. K. M. F. K. Rasel, …, M. A. Hayes. 2025. Gradient Insulator-Based Dielectrophoresis of Gold Nanoparticles. *Electrophoresis.* 46:768–776. https://doi.org/10.1002/elps.8119.

36. Barekatain, M., Y. Liu, …, M. A. Hayes. 2024. Insulator-based dielectrophoresis-assisted separation of insulin secretory vesicles. *eLife.* 13:e74989. https://doi.org/10.7554/eLife.74989.

37. Crowther, C. V., V. Sanderlin, …, G. H. Gile. 2019. Effects of surface treatments on trapping with DC insulator-based dielectrophoresis. *Analyst.* 144:7478–7488. https://doi.org/10.1039/c9an01186b.

38. Li, M., F. Feng, …, E. Song. 2022. VAMP4 regulates insulin levels by targeting secretory granules to lysosomes. *J. Cell Biol.* 221:e202110164. https://doi.org/10.1083/jcb.202110164.

39. Halban, P. A. 1982. Differential Rates of Release of Newly Synthesized and of Stored Insulin from Pancreatic Islets. *Endocrinology.* 110:1183–1188. https://doi.org/10.1210/endo-110-4-1183.

40. Gold, G., M. L. Gishizky, and G. M. Grodsky. 1982. Evidence That Glucose "Marks" β Cells Resulting in Preferential Release of Newly Synthesized Insulin. *Science.* 218:56–58. https://doi.org/10.1126/science.61815.

41. Schatz, H., C. Nierle, and E. F. Pfeiffer. 1975. Pro-)Insulin Biosynthesis and Release of Newly Synthesized (Pro-)Insulin from Isolated Islets of Rat Pancreas in the Presence of Amino Acids and Sulphonylureas. *Eur. J. Clin. Invest.* 5:477–485. https://doi.org/10.1111/j.1365-2362.1975.tb02312.x.

42. Arous, C., and P. A. Halban. 2015. The skeleton in the closet: actin cytoskeletal remodeling in β-cell function. *Am. J. Physiol. Endocrinol. Metab.* 309:E611–E620. https://doi.org/10.1152/ajpendo.00268.2015.

43. Nauck, M. A., and J. J. Meier. 2018. Incretin hormones: Their role in health and disease. *Diabetes Obes. Metabol.* 20:5–21. https://doi.org/10.1111/dom.13129.

44. Dong, T. X., S. Othy, …, M. D. Cahalan. 2017. T-cell calcium dynamics visualized in a ratiometric tdTomato-GCaMP6f transgenic reporter mouse. *eLife.* 6:e32417. https://doi.org/10.7554/eLife.32417.

45. Staton, S. J. R., K. P. Chen, …, M. A. Hayes. 2010. Characterization of particle capture in a sawtooth patterned insulating electrokinetic microfluidic device. *Electrophoresis.* 31:3634–3641. https://doi.org/10.1002/elps.201000438.

46. Schindelin, J., I. Arganda-Carreras, …, A. Cardona. 2012. Fiji: an open-source platform for biological-image analysis. *Nat. Methods.* 9:676–682. https://doi.org/10.1038/nmeth.2019.

47. Zhang, X., S. D. Carter, …, G. J. Jensen. 2020. Visualizing insulin vesicle neighborhoods in beta cells by cryo-electron tomography. *Sci. Adv.* 6:eabc8258. https://doi.org/10.1126/sciadv.abc8258.

48. Ivanova, A., Y. Kalaidzidis, …, M. Solimena. 2013. Age-Dependent Labeling and Imaging of Insulin Secretory Granules. *Diabetes.* 62:3687–3696. https://doi.org/10.2337/db12-1819.

49. Varadi, A., T. Tsuboi, and G. A. Rutter. 2005. Myosin Va Transports Dense Core Secretory Vesicles in Pancreatic MIN6 β-Cells. *Mol. Biol. Cell.* 16:2670–2680. https://doi.org/10.1091/mbc.e04-11-1001.

50. Wang, X., G. Arpino, …, L.-G. Wu. 2025. Dense-core vesicles contain exosomes in secretory cells. *Biophys. J.* 124:1747–1752. https://doi.org/10.1016/j.bpj.2025.01.003.

51. Greider, M. H., S. L. Howell, and P. E. Lacy. 1969. ISOLATION AND PROPERTIES OF SECRETORY GRANULES FROM RAT ISLETS OF LANGERHANS : II. Ultrastructure of the Beta Granule. *J. Cell Biol.* 41:162–166. https://doi.org/10.1083/jcb.41.1.162.

52. Orci, L., M. Ravazzola, …, H. P. Moore. 1987. The trans-most cisternae of the Golgi complex: A compartment for sorting of secretory and plasma membrane proteins. *Cell.* 51:1039–1051. https://doi.org/10.1016/0092-8674(87)90590-3.

53. Arvan, P., and P. A. Halban. 2004. Sorting ourselves out: seeking consensus on trafficking in the beta-cell. *Traffic.* 5:53–61. https://doi.org/10.1111/j.1600-0854.2004.00152.x.

54. Orci, L., M. Ravazzola, …, R. G. Anderson. 1986. Conversion of proinsulin to insulin occurs coordinately with acidification of maturing

secretory vesicles. *J. Cell Biol.* 103:2273–2281. https://doi.org/10.1083/jcb.103.6.2273.

55. Koch, M., L. Varela, …, T. L. Horvath. 2015. Hypothalamic POMC neurons promote cannabinoid-induced feeding. *Nature.* 519:45–50. https://doi.org/10.1038/nature14260.

56. Willms, E., C. Cabañas, …, P. Vader. 2018. Extracellular Vesicle Heterogeneity: Subpopulations, Isolation Techniques, and Diverse Functions in Cancer Progression. *Front. Immunol.* 9:2018. https://doi.org/10.3389/fimmu.2018.00738.

57. Combe, M., K. S. Isaac, …, S. Sokolenko. 2025. Quantifying extracellular vesicle heterogeneity: the effect of process conditions on protein cargo for skin therapy. *Stem Cell Res. Ther.* 16:224. https://doi.org/10.1186/s13287-025-04279-5.

58. Shaabani, N., S. R. Meira, …, M. Kulka. 2023. Multiparametric Biosensors for Characterizing Extracellular Vesicle Subpopulations. *ACS Pharmacol. Transl. Sci.* 6:387–398. https://doi.org/10.1021/acsptsci.2c00207.

59. Kravets, V., J. M. Dwulet, …, R. K. P. Benninger. 2022. Functional architecture of pancreatic islets identifies a population of first responder cells that drive the first-phase calcium response. *PLoS Biol.* 20:e3001761. https://doi.org/10.1371/journal.pbio.3001761.

60. Šterk, M., J. Dolenšek, …, M. Gosak. 2023. Functional characteristics of hub and wave-initiator cells in beta cell networks. *Biophys. J.* 122:784–801. https://doi.org/10.1016/j.bpj.2023.01.039.

61. Duh, M., M. Šterk, …, M. Gosak. 2025. Spatially bound functional heterogeneity drives modular organization in β-cell networks. *Biophys. J.* 124:3008–3022. https://doi.org/10.1016/j.bpj.2025.07.043.

Biophysical Journal, Volume *125*

Supplemental information

Dielectrophoresis reveals stimulus-induced remodeling of insulin granule subpopulations

Ashley Archambeau, Teji Korma, Aneesh Deshmukh, Mark A. Hayes, and Kate L. White

SUPPLEMENTAL INFORMATION

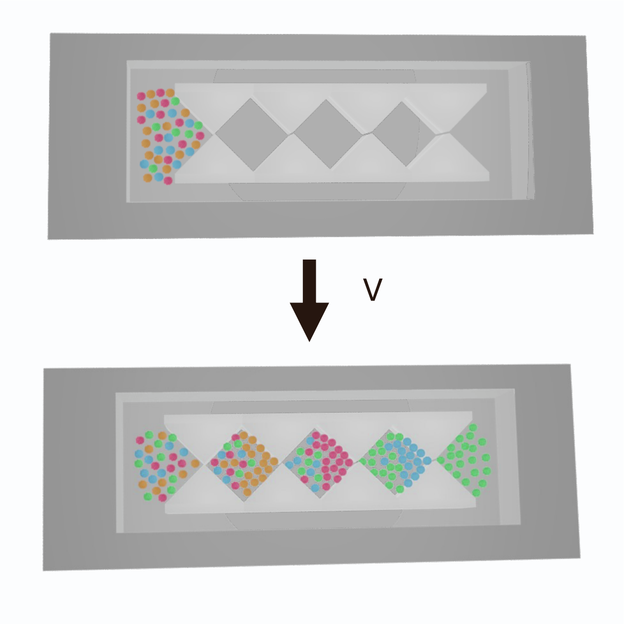

Figure S1. A truncated model of the microfluidic channel showing the separation of heterogeneous particles before (top) and after applying a voltage to separate particles (bottom).

A **Analyze intensity for each gate**

EKMr (10^9 V/m^2)	Gate	Intensity
**1.40**	**1**	0.128
**1.40**	**2**	0.0842
**1.40**	**3**	0.0202
**4.10**	**4**	0.127
**4.10**	**5**	0.0467
**4.10**	**6**	0.0520
…	…	
**22.3**	**25**	0.0343
**22.3**	**26**	0.0299
**22.3**	**27**	0.0675

B **Average replicate values**

EKMr (10^9 V/m^2)	Average Intensity	Scaled Intensity	
**1.40**	0.0775	0.284	
**4.10**	0.0753	0.275	
**6.70**	0.0602	0.217	
**9.30**	0.00428	0	**MIN**
**11.9**	0.0533	0.190	
**14.5**	0.163	0.616	
**17.1**	0.102	0.377	
**19.7**	0.262	1	**MAX**
**22.3**	0.0439	0.153	

C

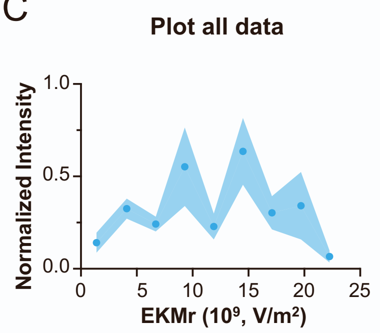

Figure S2. Example intensity processing and normalization workflow. A. The intensity of capture events is measured for each gate, with each technical replicate containing intensity measurements for each EKMr value in triplicate. Variation between these gates reflects sequential particle capture, as upstream gates are expected to capture more particles. Gates with no capture events were assigned a value of 0 before normalization. B. The intensity measurements for each EKMr value are averaged and assigned values of 0-1 according to the ratio of the intensity to the difference between the maximum and minimum intensity values. C. Each replicate is plotted to provide the typical distribution of a subpopulation. Shown here is the distribution of Rab3a at 1500 V, combining all biological replicates.

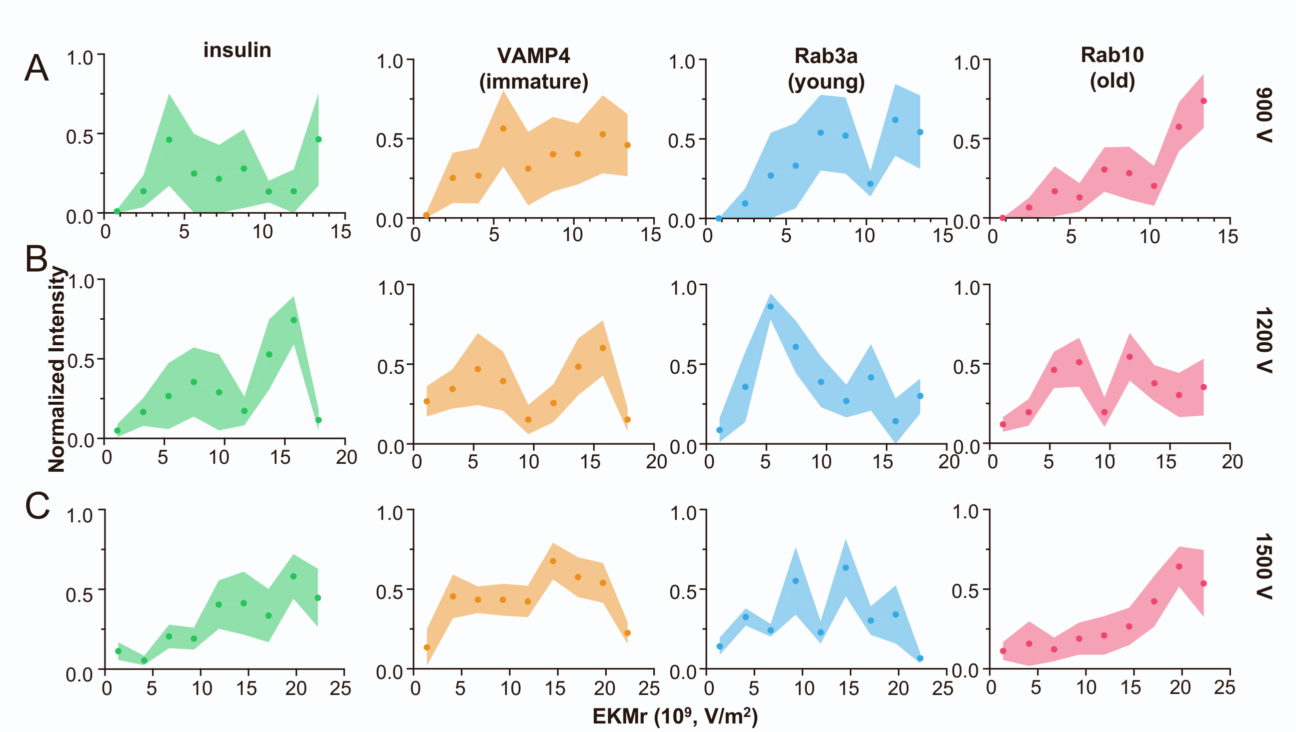

Figure S3. The EKMr distribution of different ISG subpopulations at **A.** 900 V (n=2-3 biologically independent experiments), **B.** 1200 V (n=3 biologically independent experiments), and **C.** 1500 V (n=3-4 biologically independent experiments). Values are mean ± SEM.

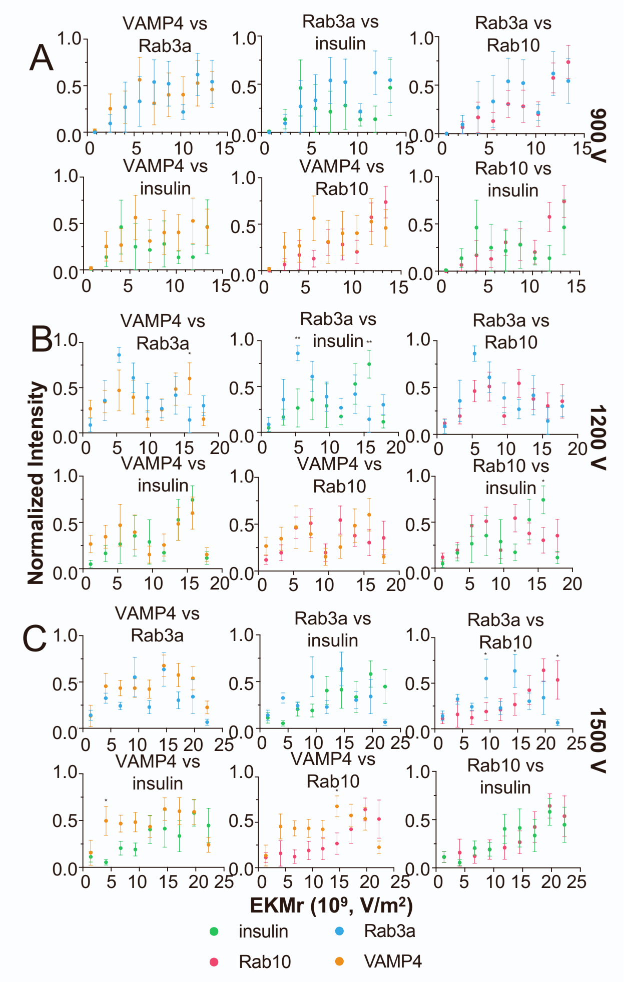

Figure S4. Comparisons of ISG subpopulation distributions at **A.** 900 V (n=2-3 biologically independent experiments), **B.** 1200 V (n=3 biologically independent experiments), and **C.** 1500 V (n=3-4 biologically independent experiments). Values are mean ± SEM (*p≤0.05, **p≤0.01 using ANOVA with Bonferroni post hoc multiple comparison correction).

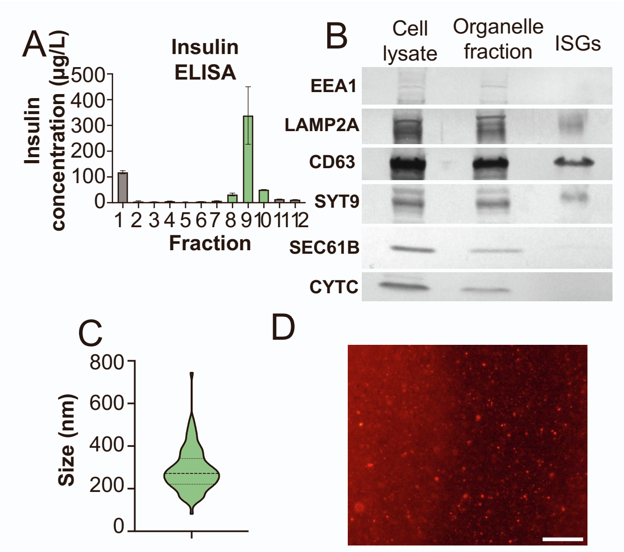

Figure S5. Validation of ISG isolation. **A.** Selection of fractions for use in separation experiments. Values are mean ± SEM. **B.** WB of cell lysate, organelle fraction, and isolated ISGs for determination of ISG purity. **C.** Size distribution of ISGs isolated from INS-1E cells. **D.** Fluorescence image of isolated ISGs. Scale bar: 250 μm.

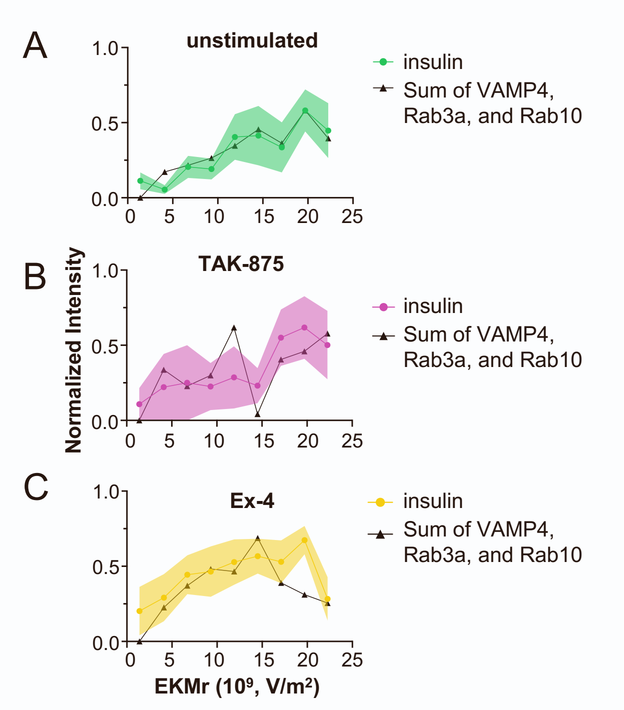

Figure S6. The cumulative subpopulation distribution approximates the total insulin distribution. For each EKMr bin, the independently normalized (0–1) intensities of VAMP4, Rab3a, and Rab10 were summed without weighting to generate a cumulative subpopulation signal. To facilitate visual comparison, this summed distribution was linearly scaled so that its maximum value matched the maximum of the corresponding insulin distribution within the same condition. Panels show **A.** unstimulated, **B.** TAK-875-stimulated, and **C.** Ex-4-stimulated conditions. Values are mean ± SEM, and lines connect points for visual guidance.

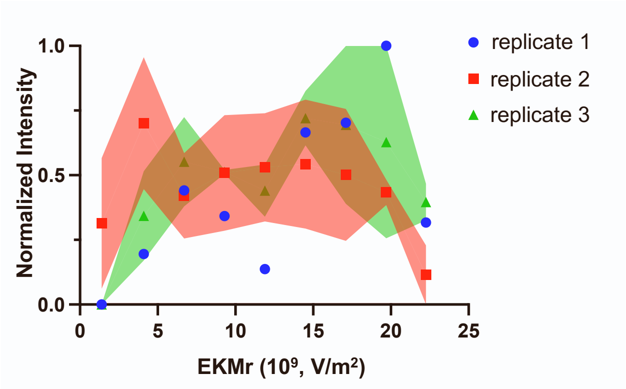

Figure S7. EKMr distributions of VAMP4-labeled ISGs separated at 1500 V from three independent biological experiments. Each replicate was independently background-corrected and scaled between 0 and 1 before plotting. Replicates exhibit similar overall EKMr profiles with expected biological variability across bins.

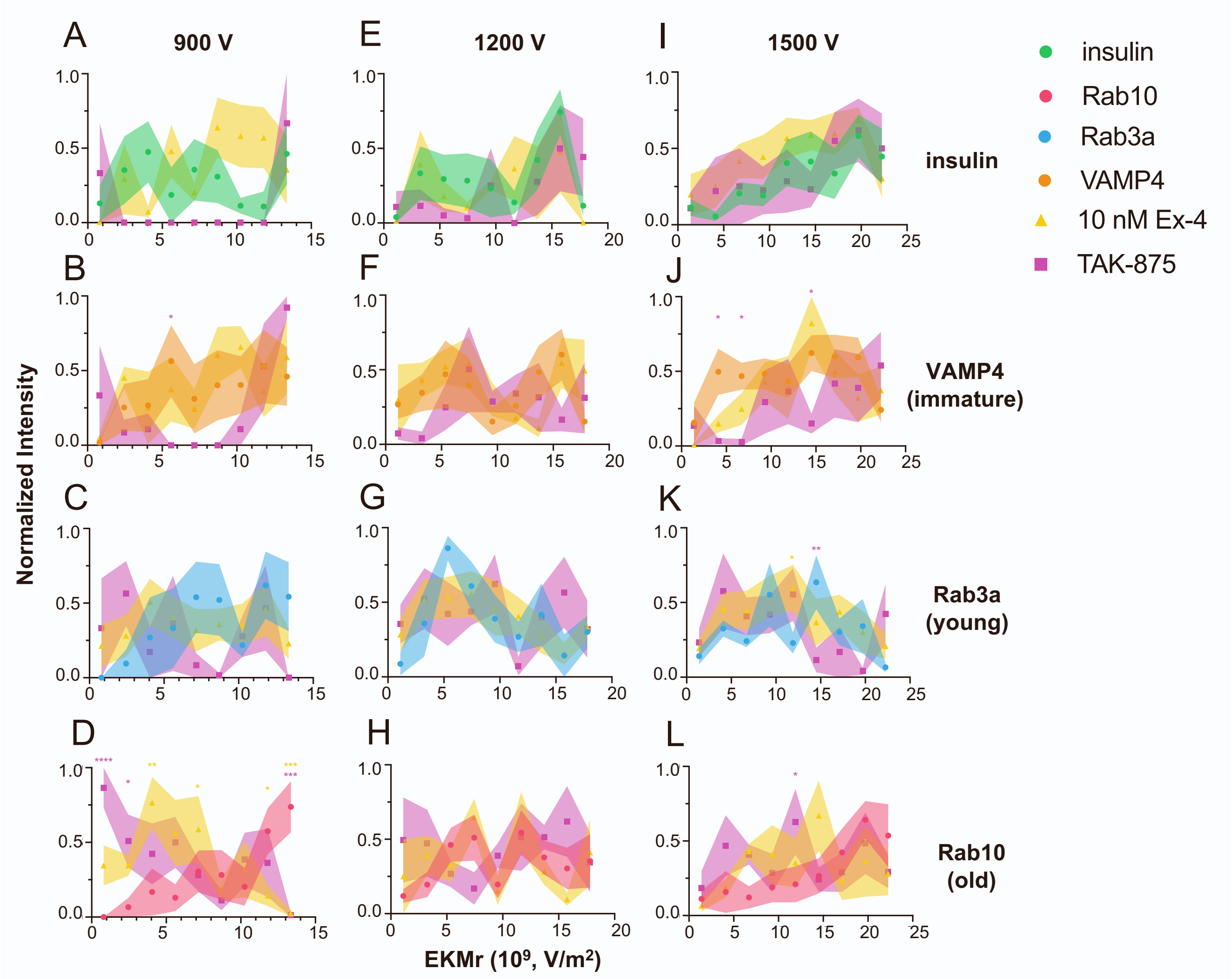

Figure S8. Changes in the EKMr distribution of each ISG subpopulation in response to TAK-875 and Ex-4 at **A-D.** 900 V (n=2-3 biologically independent experiments), **E-H.** 1200 V (n=2-3 biologically independent experiments), and **I-L.** 1500 V (n=2-4 biologically independent experiments). Values are mean ± SEM (*p≤0.05, **p≤0.01, ***p≤0.001 using ANOVA with Bonferroni post hoc multiple comparison correction. Comparisons made between stimulation and unstimulated conditions, symbols color-coded according to condition).

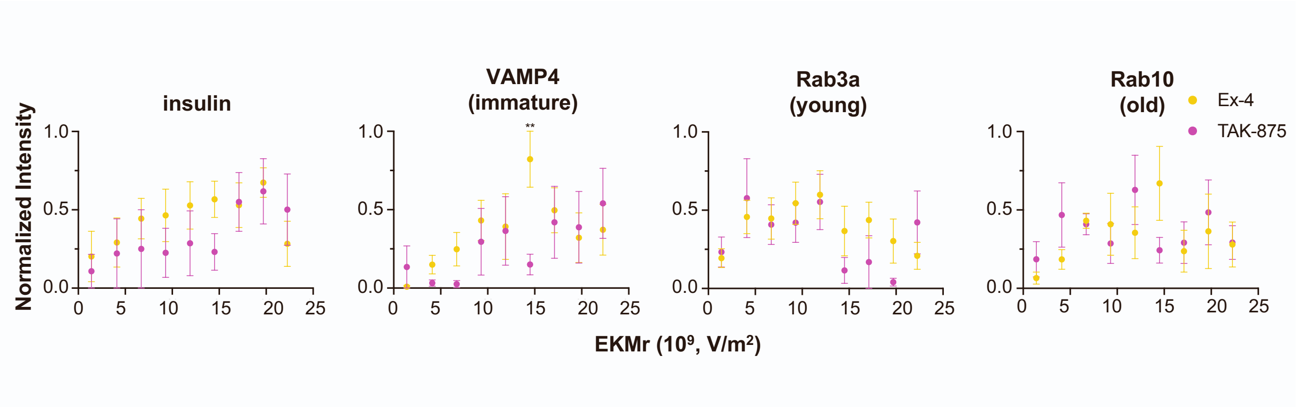

Figure S9. Differences in EKMr distributions between ISGs isolated from TAK-875 and Ex-4-stimulated cells at 1500 V (n=2-3 biologically relevant experiments). Values are mean ± SEM (*p≤0.05, **p≤0.01, ***p≤0.001 using ANOVA with Bonferroni post hoc multiple comparison correction).

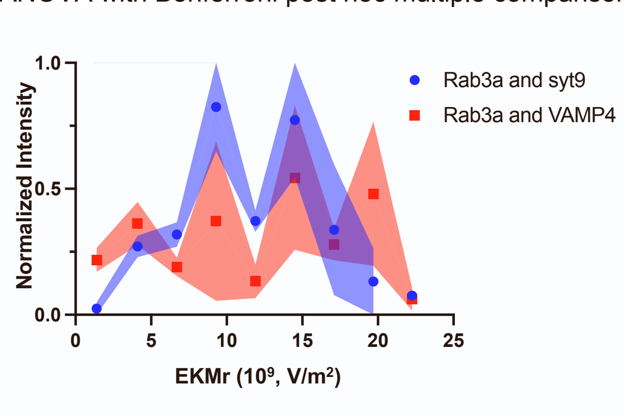

Figure S10. The EKMr distribution of young ISGs at 1500 V is not significantly affected by the differences in a second antibody label (syt9 or VAMP4, n=1-2 biologically independent experiments). Values are mean ± SEM.

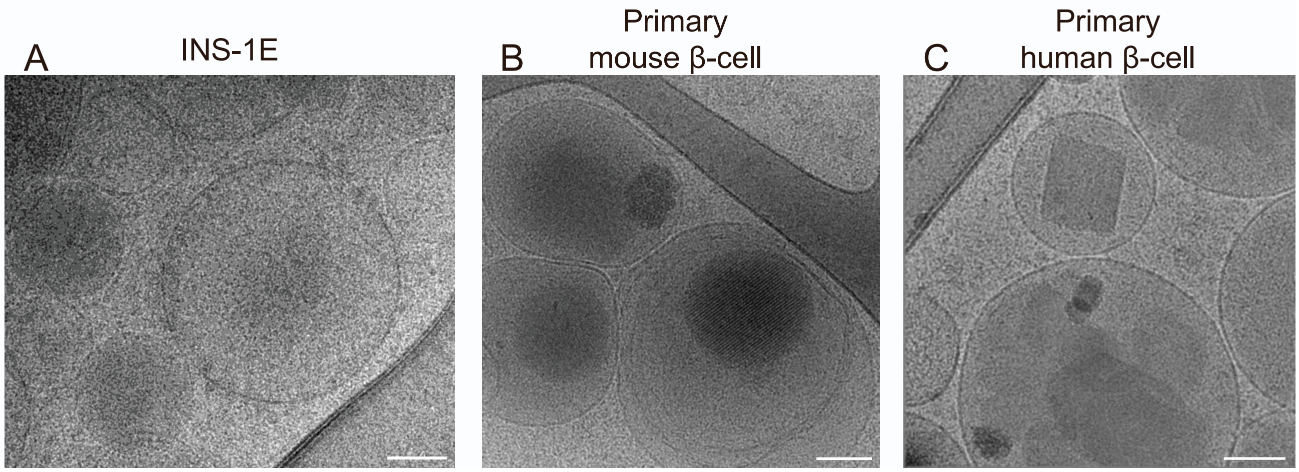

Figure S11. CryoET slices (**A-B**) and cryoEM images of ISGs inside INS-1E, primary mouse β-cells, and primary human β-cells. Magnification: **A-B.** 26,000x, **C.** 92,000x. Scale bar: 100 nm. A Gaussian blur of 1.0 nm was applied for clarity.

Table S1. Statistical comparisons between markers in the unstimulated condition.

Table S2. Statistical comparisons between the unstimulated condition and either the TAK-875 or Ex-4 condition.

Table S3. Statistical comparisons between the TAK-875 and Ex-4 conditions at 1500 V.

Video S1. Sample video of data collection of VAMP4-labeled ISGs separated in a microfluidic channel at 1500 V. Scale bar: 250 μm.
